# Religious parents receive more alloparental aid in rural Bangladesh

**DOI:** 10.1017/ehs.2025.10029

**Published:** 2025-12-12

**Authors:** Theodore Samore, Richard Sosis, John Shaver, Radim Chvaja, Matthew Conrad, Anushe Hassan, Robert F. Lynch, Susan Schaffnit, Laure Spake, Joseph Watts, Mary K. Shenk, Rebecca Sear, Nurul Alam

**Affiliations:** 1 Religion Programme, University of Otago, Dunedin, New Zealand; 2 Department of Anthropology, University of Connecticut, Storrs, USA; 3 Department of Anthropology, Baylor University, Waco, USA; 4 Faculty of Economics, European Research University, Ostrava, Czech Republic; 5 Department of Population Health, London School of Hygiene and Tropical Medicine, London, UK; 6 Independent researcher, USA; 7 Washington State Department of Social and Health Services, Olympia, WA, USA; 8 Department of Anthropology, Binghamton University (SUNY), Binghamton, NY, USA; 9 School of Psychology, Speech and Hearing, University of Canterbury, Christchurch, New Zealand; 10 Department of Anthropology, The Pennsylvania State University, University Park, PA, USA; 11 Centre for Culture and Evolution, Brunel University, London, UK; 12 ICDDR, B: Centre for Health and Population Research – Health Systems and Population Studies Division, Dhaka, Bangladesh

**Keywords:** allocare, religion, cooperation, signalling

## Abstract

Researchers have long speculated about the evolutionary benefits of religiosity. One explanation for the evolution of religious ritual is that rituals signal commitment to co-religionists. As a major domain of prosocial behaviour, alloparental care – or care directed at children by non-parents – is a plausible benefit of religious signalling. The religious alloparenting hypothesis posits that parents who signal religious commitment receive greater alloparental support. Prior research on religiosity, cooperation, and allocare tends to treat individuals as isolated units, despite the inherent collective nature of religious cooperation. Here, we address this limitation in a survey-based study of 710 parents in rural Bangladesh. Instead of focusing only on mothers, we consider the interplay between both mothers and fathers in eliciting allocare, and leverage variation in the covertness of religious rituals to test a key mechanistic assumption linking religious ritual with cooperation. We find that parents who practice religious rituals more frequently receive greater alloparental support from co-religionists. This effect is moderated by parent gender, as well as variation in the visibility of religious rituals. Women’s private practices positively affect only those alloparents with whom they share a household, whereas men’s public practices positively affect alloparents more broadly.

## Graphical abstract

**Figure:**
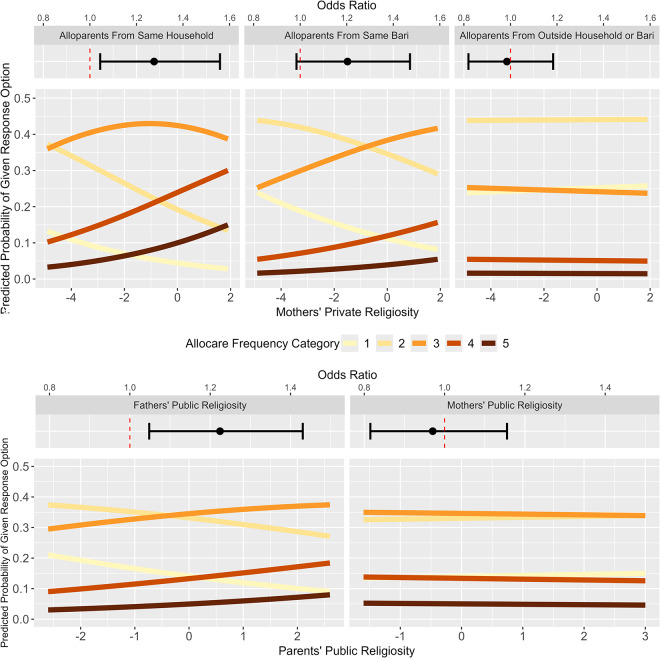

## Social media summary

Men and women in rural Bangladesh use gender-specific kinds of religious practices to elicit more alloparental support from their religious networks.

## 1. Introduction

The *costly signalling theory of religion* (Bulbulia, 2004; Sosis & Alcorta, 2003) posits that religious rituals may have in part evolved as costly signals of group commitment. Given the threat of exploitation (Dunbar, 1999), cooperation and collective action may be more stable in large human groups (as compared to the smaller groups which likely characterized our ancestors) when individuals can reliably, and honestly, signal group commitment (Dunbar & Sosis, 2018; Lang *et al.*, 2022). Religion, and religious rituals, may be particularly well-suited to scaffolding the trust required for large-scale cooperation (Sosis, 2005), although the precise importance of costliness in maintaining those signals may vary (Barker *et al*., 2019). Specifically, as laid out by the costly signalling theory of religion, because of their costs, religious rituals may honestly signal group commitment, and thus the suitability of an individual as a cooperative partner for other members of the religious community (Sosis, 2003). Individuals who engage in frequent religious practice and ritual are both more likely to engage in prosociality themselves, and also more likely to receive cooperation from co-religionists (Power, 2017; Ruffle & Sosis, 2007; Soler, 2012; Sosis & Bressler, 2003). Although cooperation can occur across many different domains, cooperative alloparenting is a human universal (Kramer, 2010; Sear & Mace, 2008), and hence a likely target for mutual aid between co-religionists.

At the same time, religion has been observed to have relationships with fertility and offspring investment. Religious parents simultaneously tend to have high fertility (Blume, 2009; McQuillan, 2004), yet their children do not seem to experience the declines in children’s embodied capital that tend to be associated with higher fertility (Bartkowski *et al.*, 2008; Ellison & Xu, 2014; Lawson & Borgerhoff Mulder, 2016). This paradox of religious fertility (Shaver *et al.*, 2019) may be resolved by the putative cooperative benefits of religiosity (Norenzayan *et al.*, 2016; Purzycki *et al.*, 2016; Purzycki & Sosis, 2022). That is, highly religious parents may be able to mitigate the costs typically associated with high fertility by leveraging benefits from co-religionists in the form of alloparental support. Although this possibility has yet to be empirically tested, the broader literature on cooperative reproduction in humans indicates that nonmaternal care can have positive, context-specific effects on both child outcomes (Bell *et al.*, 2013; Hrdy, 2007; Kramer, 2010). Given that cooperative childrearing appears to be a human universal (Brown, 1991; Sear & Mace, 2008), and if religion can intensify aid among practitioners, shared religious affiliation may thus be one mechanism through which parents access the alloparental care their children need, or access higher amounts of alloparental support generally.

The *religious alloparenting hypothesis* (Shaver *et al.*, 2019) predicts that parents who signal group commitment through religious behaviour will receive more alloparental support from co-religionists, which in turn may ameliorate the tradeoff between family size and per-child investment. The costly signalling theory of religion explains why higher religiosity may result in receiving more support from co-religionists, whereas the religious alloparenting hypothesis extends the former into the domain of cooperative childrearing and ways in which that support can mitigate embodied capital tradeoffs. The hypothesis thus addresses and ties together two important and longstanding questions: did religion evolve because of its cooperative affordances, and do those affordances extend to the domain of child rearing?

The religious alloparenting hypothesis has already been tested in several different contexts. For example, in a sample of English mothers, church attendance was associated with larger social networks, and more aid from co-religionists (Shaver *et al.*, 2020); in New Zealand, more religious people were more likely to provide allocare (Shaver *et al.*, 2019) and to have higher fertility; and in an online sample of Americans and British, more religious women received more allocare from non-partner kin (Spake *et al.*, 2024). However, this prior research is limited in several ways.

First, prior research has only considered the role of mother’s religiosity in eliciting childcare from alloparents. In the cooperative reproduction literature, allocare is typically framed as being directed at mothers and their offspring (Bentley & Mace, 2012; Page *et al.*, 2019, but see Micheletti *et al.*, 2022). However, allocare entails inclusive fitness benefits not just for mothers, but also for fathers, and allocare ought to be considered as having distributed benefits across different levels of the family unit. More broadly, the literature on religion and human cooperation often focuses on dyadic interactions between individuals. Yet, given that humans are a socially complex species, support is often organized, distributed, and received at the family level. It is misguided to search for the social effects or benefits of phenomena such as religious cooperation and yet treat individuals as independent from their families or other units of social organization. In the context of cooperative reproduction, this can be addressed by studying the effects of religiosity on allocare at the familial, rather than individual, level.

In the context of the religious alloparenting hypothesis, the elicitation of alloparental care may be shaped by the religiosity of both parents. That is, when making decisions about investment levels, alloparents may consider the religiosity of both parents. In order to fully assess the religious alloparenting hypothesis, it is critical to examine the father’s religiosity in addition to the mother’s. Yet, the mother’s and father’s religiosity may not be equipotent in their elicitation of allocare, and the relative contributions of each may be contingent on varying sociocological factors. For example, if religious ritual in part signals one’s suitability as a cooperative partner, alloparents who provision resources may attend most strongly to the religiosity of the parent who controls how that resource is used within the household, or the parent who would make future decisions about reciprocating the resources. Religiosity is also overdetermined (e.g. Major-Smith *et al.*, 2023), and likely serves additional functions beyond eliciting allocare (Purzycki & Sosis, 2022). If these functions differ between women and men in some socioecological contexts, then the relationship between alloparental support and religiosity may vary across genders as well. The parent-directed benefits of allocare may also differ based on society-specific gender norms and other factors. For example, in societies where women engage in more domestic labour and men engage in more wage labour, direct allocare (e.g. physically caring for a child) may proximately benefit women more than men by freeing up their domestic labour, whereas indirect allocare (e.g. providing resources) may have relatively higher benefits for men by indirectly increasing their income.

The second limitation is that the religious alloparenting hypothesis makes a causal, evolutionary argument linking the practice of religious ritual with increased child-rearing support. The hypothesis predicts that when parents engage in religious ritual, it induces co-religionist alloparents to engage in more care, given that the ritual serves as an honest signal of the parents’ own commitment. This implies a crucial proximate mechanism – observing religious rituals provides co-religionists with the most accurate information regarding the frequency with which they are practised. If ritual behaviour is non-observable by a potential cooperator, it contains less signal value and may not affect the assessment of an individual’s religiosity and group commitment. As an important condition for the religious alloparenting and costly religious signalling hypotheses, the observability of ritual behaviour provides an opportunity to test the causal and adaptationist assumptions made by those frameworks (Laland *et al.*, 2011). However, we note that direct observation is not the only mechanism for acquiring information about an individual’s religiosity. If a potential cooperator does not witness ritual behaviour first-hand, they may nevertheless have access to reputational information from second-hand parties regarding an individual’s religiosity, although this should weigh less heavily than directly observed behaviour.

Although prior research has found support for both theories (e.g. Shaver *et al.*, 2019, 2020; Soler, 2012; Sosis & Bressler, 2003), there has been little work testing whether the empirical results are consistent with the requisite mechanisms. For example, an alternative possibility is that parents who receive more alloparental support may have more time and resources to engage in religious behaviours, which may have other downstream benefits. As explored above, an individual’s reputation for religiosity may also be a useful proxy for their commitment to religious practices. In these cases, religious rituals may be connected or unconnected to allocare irrespective of whether they are private or public. On the other hand, evidence that the observability of religious ritual regulates the elicitation of care would provide support for the causal pathway proposed by the religious alloparenting and costly religious signalling hypotheses.

The current study addresses the above limitations. We collected data from hundreds of households in rural Matlab, Bangladesh, a region with highly religious – and religiously diverse – communities of Muslims and Hindus (Lynch *et al.*, 2022). We are able to evaluate whether parents who engaged in more religious rituals and behaviours received more alloparental support for their children, disambiguating the relative contributions of both mother’s and father’s religiosity to allocare. In order to test the causal assumptions of the religious alloparenting hypothesis, we assessed whether the visibility of various religious rituals moderated the association between parent religiosity on alloparents.

## 2. Research questions

*RQ1. Does the mother’s and father’s religiosity associate with alloparental support?*

First, we sought to test the primary prediction of the religious alloparenting hypothesis: do more religious parents receive care more frequently from alloparents in their religious community? We also tested whether the father’s and mother’s religious practices were equally important for eliciting allocare. Because the importance of mother’s versus father’s religiosity in eliciting allocare is likely sensitive to features of the cultural environment as well as other conditional factors, we did not make strong directional predictions about their relative effects. Instead, this study sought to document the pattern of association between religiosity and allocare as a function of parent identity. The relative effects of mother’s and father’s religiosity on allocare will shed light on the generalizability of the religious alloparenting hypothesis across gender roles.

The mother’s and father’s religiosity could affect allocare in several different configurations. First, the effects of their religiosity on allocare may be independent, such that one does not affect or interact with the other. In this case, both parents’ religiosity may independently elicit allocare, or just one parent’s, or neither’s. For example, the effect could be gender-dependent if only mother’s or only father’s religiosity is relevant for alloparents making investment decisions. Second, allocare may intensify when both parents are highly religious, relative to when only one parent is highly religious. If mother’s and father’s religiosity independently lead to larger networks of alloparents, and/or if potential alloparents attend to the religious commitments of both parents, then the relationship between religiosity and allocare should be highest when both parents are devoutly religious. Correspondingly, if one parent is devout and the other is not, allocare may be intermediate, while allocare may be lowest when neither parent is strongly religious. Alternatively, there may not be intensification of allocare when both parents are jointly highly religious. For example, potential alloparents may employ a heuristic of attending to the religiosity of the most or least religious parent, regardless of the other parents’ religiosity. Third, there could be an interaction between mother’s and father’s religiosity, such that the effect of one is contingent on the level of the other.

We also tested whether the effects of parents’ religiosity on allocare generalized across other features of the alloparental system, notably the ages of the focal children, whether the allocare in question was indirect or direct, and the identities of the alloparents. For example, matrilateral relatives may attend more to the religiosity of the father, which may serve as a signal of not just religious commitment, but paternal commitment as well (Xygalatas *et al.*, 2022). These factors shed light on the generalizability of the putative effect across diverse conditions. Because the sample comprised both Muslims and Hindus, we can compare results across two different religious traditions in which ethnicity is held constant.

*RQ2. Does the visibility of religious rituals moderate the effects of parental religiosity on allocare?*

The religious allocare hypothesis posits that religious practices and rituals may in part serve as signals that elicit alloparental support from co-religionists. If this is true, then the effect of parental religiosity on allocare should be sensitive to the observability of the parent’s ritual practice by potential alloparents. That is, when rituals are highly visible, religiosity should elicit more alloparental care; however, if some rituals are unobserved by the potential alloparent, then there should be no association. If the effect of parents’ religiosity on allocare is indeed sensitive to these factors, it would provide evidence for the proximate mechanism described by the costly signalling theory of religion.

We can test this prediction in the current study by leveraging the heterogenous nature of both religious rituals and the physical proximity between parents and alloparents. Rituals vary in their location of practice, with some taking place publicly within the religious community (e.g. praying in a mosque, attending worship services at a temple), whereas others take place privately within the home (e.g. private prayer, performing worship at home). Potential alloparents vary in how likely they are to observe those practices depending on how closely they reside to the parents in question. For example, alloparents living within the same household as the parents and focal child (e.g. siblings or grandparents of the focal child) may have access to information about both private and public religious practices. In contrast, alloparents living outside the focal child’s household may only regularly observe the parents’ public practice. In the community of Matlab, Bangladesh, there is also a third, intermediate category organized around the *bari*, or a neighbourhood of small houses containing extended patrilocal families (Shenk *et al.*, 2013). *Bari* members may have more insight into parents’ private religious practices than co-religionists outside the *bari*, but less than household members from the same household.

We tested whether parental private religious practices would elicit greater allocare from same-household and same-*bari* alloparents but not from alloparents outside the household and *bari*, consonant with the mechanistic logic presented above. By contrast, we assessed whether public religious practices would elicit greater care from all alloparents, given the greater observability of those public rituals. This pattern of results would support the causal argument that greater religiosity – in the form of ritual practice and other observable forms – elicits more alloparental support from co-religionists.

## 3. Methods

### 3.1. Project overview

This study was part of a broader project on the evolutionary dynamics of religion, family size, and child success across five global fieldsites. The present study focuses on data collected between 2022 and 2023 in Matlab, Bangladesh. Focusing on one site allows us to unpack the mechanisms operating in a specific cultural context in more detail. Further, information on father’s religiosity was only collected in three of the study sites, and of those three, only two had full data on parents’ allocare networks. The overall project was pre-registered prior to data collection, and the present study was pre-registered before any analysis was conducted, due to data being collected before this specific analysis was conceived. This study was approved by the Ethical Review Committee of the International Centre for Diarrhoeal Disease Research, Bangladesh (icddr,b), which has oversight over the Matlab fieldsite, as well as the University of Otago Human Ethics Committee and the Institutional Review Board of the Pennsylvania State University, and all methods were performed in accordance with relevant guidelines and regulations. Informed consent was obtained before participation. The complete questionnaires in English and Bengali, pre-registrations, analysis code, and the anonymized data required to replicate the analyses are available at https://osf.io/b865v/.

### 3.2. Participants

Participants were recruited in from the Matlab Health and Demographic Surveillance System fieldsite run by the icddr,b (Alam *et al.*, 2017), located in Matlab, Dokhin upazila, Bangladesh. The fieldsite includes 142 villages with approximately 230,000 residents. Residents are ethnic Bengalis, and split approximately between Islam (88.2%) and Hinduism (11.8%) (Alam *et al.*, 2017). Economic activity is divided between intensive agriculture, fishing, and wage labour, and individuals primarily live in patrilocal households that are organized into extended patrilateral neighbourhoods called *bari*. Among individuals from both traditions, religion tends to be central to most people’s lives (Devine *et al.*, 2019). Both men and women participate in religious life, although women are unlikely to work outside the home, and more likely to practice religion privately. Crucially, engagement in religious practice and ritual varies substantially across individuals (Lynch *et al.*, 2022).

The data are drawn from a full population census held by icddr,b. The sample was stratified by religion, and half of the women were sampled from each religion. Within religions, women were sampled randomly from among women with at least once child. The resulting sample contains 1,003 women with children. Women completed an interview-based survey, which included batteries of questions regarding their religiosity, fertility, and alloparental support alongside numerous other topics. For each woman, where applicable, three focal children were chosen at random from among her offspring, which were categorized into three age groups; 1–5, 6–16, and 16+. Women then named all the individuals in their social network who provided allocare for each focal child, and indicated how frequently different kinds of allocare were performed for a focal child. Of the 1,003 mothers in the sample, 705 of their husbands were also interviewed, and answered questions about their own religiosity. Husbands were not interviewed if they were working as labour migrants in another part of Bangladesh or abroad, dead, divorced from the mother, or otherwise unavailable.

For the present study we selected focal children based on the following criteria. First, they had to belong to either the young (ages 5 and under) or middle (ages 6–16) age groups, because we were interested in the effects of parent religiosity on direct allocare. Children aged 16 and older were excluded. Second, the focal children had to live in the same household as both their parents, as the relative effects of mother’s and father’s religiosity – and the effects of alloparent proximity to those parents – were being assessed. Third, the focal children’s fathers had to have completed the survey in order to have data on the father’s religiosity. Fourth, the alloparents had to share the same religion as the parents, because we were testing the hypothesis that religiosity elicits greater alloparental support from co-religionists. Of the alloparents reported by mothers, 99% shared the same religion as the parents they helped. This resulted in a final sample of 455 children from 305 households/spousal pairs, with 1,115 alloparents having provided those children with care.

### 3.3. Measures

Prior to data collection the following measures were pre-tested and refined according to feedback from focus groups and community interviews. Participants were very familiar with the concept of allocare, which is common in local communities, and also quite familiar with the concept of religiosity as a trait that varies across individuals in the local context (Lynch *et al.*, 2022).

**Allocare**: Allocare was measured using the following procedure. First, mothers were asked to list all individuals who provided direct care for the focal child in the last week (e.g. supervising the child, playing with the child, and other forms of direct interaction), as well as all individuals who provided provisioning care for the focal child in the last three months (e.g. providing money or resources). To be included, prospective alloparents had to provide either direct or indirect allocare, but some provided both. For each alloparent thus identified, mothers were then asked to indicate how often that alloparent provided allocare across a set of prespecified frequencies, using ordinal Likert scales. For direct allocare, mothers were asked how frequently the alloparent had cooked for, disciplined, and supervised the focal child in the last two weeks (6-point scale from ‘never’ to ‘multiple times per day’). For young focal children (age < 6), there was an additional item concerning how frequently the alloparent had washed the child. For indirect allocare, mothers were asked how frequently in the last three months the alloparent had paid for medical expenses, provided food, money, or gifts, and taken the focal child shopping (5-point scale from ‘never’ to ‘more than five times’).

Because the allocare items were measured on an ordinal scale, the differences between the successive categories were not necessarily equal in magnitude. Simply summing the allocare items to obtain the total amount of allocare received per focal child would require naively assuming that those magnitudes were equal (Liddell & Kruschke, 2018). Likewise for averaging the items to calculate the average amount of allocare per alloparent. Instead, the allocare items could be modeled as an ordinal outcome, where higher categories indicated that the particular category of allocare (e.g. supervision) was performed more frequently, and with each instance of allocare treated as its own response. The advantage of this approach is that it most closely corresponds with the structure of the data, and does not require erroneously treating ordinal data as metric. In addition, analysing the data at the level of individual acts of allocare allows us to incorporate information about alloparent and allocare characteristics within the models, including household proximity.

However, this approach has disadvantages as well. First, although the ordinal categories corresponded with how frequently care was provided in specific categories by specific alloparents, we could not estimate the total amount of allocare received by focal children given that the ordered frequency responses could not be summed. Second, alloparents provided on average 2.34 care acts (SD = 1.29), out of a total of six to seven depending on the age of the focal child. As a result, most allocare ratings were zero. The processes that generated the presence or absence of a specific type of allocare for a given alloparent were plausibly different than those that contributed to the frequency when at least some care was provided. For example, characteristics of the alloparent (e.g. gender or age) may influence what kinds of care they provided, but not the frequency at which they performed the types of care they did provide. Therefore, given this zero-inflation, we considered the presence or absence of a particular type of allocare to be separate from the frequency at which particular types of allocare were performed.

Considering these challenges, we chose to operationalize alloparental care as follows: when providing care, how often did an alloparent perform that type of assistance as measured on an ordinal scale? We hypothesized that, all else equal, when providing allocare, the alloparents of more religious parents would provide that care more frequently.

Alternative outcomes were also considered. As detailed above, we separated the presence or absence of particular types of allocare from the frequency at which they were performed. However, it is not clear whether performing a more diverse range of care acts constitutes higher investment by an alloparent independent of frequency. Therefore, we consider this outcome exploratory, and results are reported in the Supplement (see page S34). In addition, although there were principled reasons to exclude zeroes from the ordinal outcome, we tested whether results were sensitive to their inclusion.

Finally, we analysed the number of alloparents per focal child. However, it is not clear whether the size of an alloparent network corresponds with the total care received by a focal child. For example, smaller networks with higher investing alloparents may provide more total benefit to parents and their children. Therefore, we considered this outcome exploratory, because the allocare frequency outcome described above corresponds more closely with the actual amount of care being directed at the focal children.

**Religiosity**: The mothers and fathers of the focal children independently answered survey questions regarding their own religiosity. Some of these questions were shared across all participants (e.g. ‘How many times in the past 4 weeks did you attend temple/mosque for prayer or to listen to religious discussion, etc.?’), whereas others were specific to either Muslim (e.g. ‘Do you perform the obligatory fasting during the month of Ramadan?’) or Hindu (e.g. ‘Do you participate in Ekadosi fasting?’) participants. Some variables were measured on an ordinal scale, some variables were counts of the presence or absence of particular behaviours, and finally some were open-ended frequency measures. For purposes of scale development, ordinal variables were treated as ordered factors, and counts were log-transformed. Multiple imputation using the *mice* package (van Buuren & Groothuis-Oudshoorn, 2011) in R was conducted for missing items (0.5% of responses were missing across the religiosity items). See page S3 in Supplement for details on all religiosity items included in the survey, as well as descriptive plots of the constituent variables.

Because expressions of religiosity differ between Islam and Hinduism, religiosity scores were computed separately for participants from the two religions, using all adult participants from the data set. Moreover, religious expression in both religions also varies between men and women, hence religiosity scores were calculated separately by gender as well. For each of the four combinations between gender and religion, the following procedure was used to calculate public and private religiosity.

First, we examined the distribution of responses for each religiosity item. Many items showed essentially no variation across participants; for example, 100% of Muslim fathers reported that religion was ‘very important’ to how they saw themselves. Items that lacked variation were then dropped.

Second, the remaining items were then sorted into one of three categories: public religious rituals, private religious rituals, and subjective self-assessments of religiosity. Public religiosity was defined as behaviours that could be plausibly observed in public by other members of the religious community. For example, how many times the participant had attended religious services in the last month. Private religiosity was defined as behaviours that were reported to occur in the home, such as the recitation of religious texts. Subjective religiosity was comprised of participants’ own assessments of their religiosity, such as the importance of religion in one’s life, and thus did not include actual behaviour or practice. Because this study was concerned with how ritual acts may have influenced potential alloparents, we focused on practices that were ritualized and informationally accessible to potential observers, and thus subjective religiosity items were not included in the main analyses.

Third, in order to ensure that the items designed to measure private and public religiosity accurately captured the underlying latent traits, we performed principal components analyses on the public and private religiosity items respectively (see Supplement page S3). These factors explained between 39% and 56% of the variance, depending on the religion, gender, and type of religiosity in question.

Fourth, principal component scores were then extracted, standardized, and merged into a combined database across all genders and religions, with two religiosity scores corresponding to private and public religiosity respectively. Note that the religiosity composites were standardized within gender and religion groupings. For example, if a Hindu mother’s private religiosity score was one, that would indicate that she was one standard deviation more privately religious than the mean private religiosity among Hindu mothers in the sample. Standardizing within groups ensured that we could assess the effects of religiosity on allocare relative to otherwise similar members of the community (i.e. from the same religion and gender).

One trade-off of this approach with principal component analyses is that religiosity cannot be compared in an absolute sense, since component scores are relative within gender and religion groupings. Examining the descriptive plots of the underlying religiosity variables (see Supplement page S7), men tended to engage in fewer private practices than women, but more public practices. Patterns were relatively consistent across religions, although there were a subset of Muslim fathers who reported attending Mosque dozens of times a week, with no commensurate pattern among Hindu fathers attending Temple.

**Figure 1.**
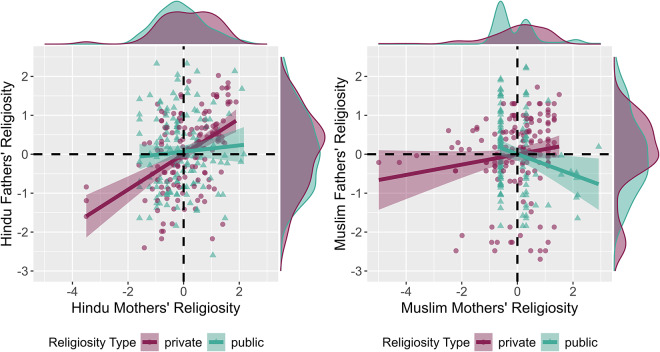
**Relationship between mother’s and father’s religiosity.** Scatterplot of raw data (dots) with fitted regression lines and 95% confidence intervals (shaded intervals) showing the relationship between mother’s and father’s private and public religiosity, disambiguated by religious affiliation. Hindu parents are on the left, and Muslim parents are on the right. Density plots along the *x*- and *y*-axes show the distribution of religiosity scores. Mother’s and father’s religiosity scores are standardized within religion and gender (e.g. a score of one would indicate religiosity one standard deviation higher than other same-gender, same-religion participants).

Finally, an individual’s private religiosity was modestly correlated with their public religiosity (mothers: *r* = .14, *p* = .001; fathers: *r* = .36, *p* < .0001). However, within spousal pairs, husbands’ and wives’ religiosity had variable associations. First, husbands’ and wives’ levels of private religiosity were correlated among both Hindus and Muslims (*r*s = .13–.41, *p*s = < .0001–.027), although the effect was stronger for the former. Second, although spouses levels of public religiosity were positively correlated among Hindus (*r* = .13, *p* = .027), there was a negative association among Muslim spouses (*r* = −.16, *p* = .008). Third, across both religions, there were no significant associations between spouses levels of public and private religiosity (*r*s = −.05–.12, *p*s = .051–.554). See Figure 1 for visualization of religiosity scores and their correlations, disambiguated by religious affiliation.

**Covariates**: Based on the results of a directed acyclic graph (see Analytical Strategy section), five covariates were also included in the regression models. These were comprised of mother’s and father’s ages, mother’s and father’s secular education, and families’ socioeconomic status (SES). SES was operationalized using a composite variable derived from a principal component analysis of mother’s assessments of how wealthy, educated, and high or low status their families were relative to their neighbours as a child; assessments of how wealthy and high or low status their families were relative to their neighbours at the time of data collection; and objective measures of household wealth and status at the time of data collection. See page S7 in Supplement for details and descriptive plots. Note that we did not have data on father’s impressions of past or current SES.

**Moderators**: The primary moderator of interest was alloparent proximity, in order to test whether the private versus public nature of various religious practices influenced allocare. This was operationalized as an index of alloparents’ proximity relative to the focal child: whether the alloparent lived in the same household as the focal child, the same *bari*, or in a different household/*bari*. Additional moderators were added in some models in order to test the robusticity of the main results. These included the focal child’s age group (young or old), the parents’ religion, the allocare category (direct versus indirect), the specific allocare type (e.g. supervising the child, providing resources for the child, etc.), the alloparent’s gender, and the alloparent’s relationship in respect to the focal child (matrilateral kin, patrilateral kin, sibling, and non-kin).

## 4. Results

### 4.1. Analytical strategy

Prior to conducting analyses, we pre-registered a Directed Acyclic Graph (DAG) to represent the assumed causal structure of the hypothesized relationships, in order to identify whether any covariates could potentially confound the assessment of a relationship between parent religiosity and alloparent support (see Figure S8 in Supplement). The directed acyclic graph indicated that in order to unbiasedly assess the direct relationship between parent religiosity and alloparental care, the following covariates should be accounted for: parent education, parent age, and parent SES.

Our primary goal was to assess whether parents’ religiosity positively covaried with the amount of alloparental care the parents’ children received, as a function of the observability of parents’ religious behaviours to co-residing and non-co-residing alloparents, respectively. As detailed in the Methods section, the estimand was operationalized by measuring how frequently a given type of allocare was provided, conditioned on that type of allocare being performed at least once within the specified time frame. This outcome was used to assess whether, all else equal, when engaging in allocare, the alloparents of more religious parents provided that help more frequently.

To test this hypothesis, we fit a series of cumulative link mixed models, given that the allocare frequency items had been rated on an ordinal scale. See Supplement page S18 for a general equation for the cumulative link mixed models utilized. First, the child-level data were lengthened such that there was a single allocare frequency outcome variable, with each row corresponding to every instance any type of allocare had been reported as having been provided for a focal child. Therefore, the number of observations (*N* = 3,475) corresponded to the number of focal children, times the number of alloparents each focal child had, times the number of types of allocare each individual alloparent provided at least once during the specified time frame. Index variables indicated the spousal pair, the focal child, and the specific alloparent for each allocare observation. These were included as random effects in subsequent models. Factor variables were also included for the specific allocare type (e.g. supervision, resource provision, etc.), allocare category (direct or indirect), focal child age group (under five versus over five), alloparent identity (e.g. matrilateral, non-kin, sibling, etc.), and finally the proximity of the alloparents to the parents (living in the same household, living in the same *bari*, or living in a different household and *bari*). These were used to test the robusticity of the main results. Table 1 provides a list and brief description of every variable that was used in at least one model. See Figure S9 in Supplement for zero-order correlations between the covariates included in the cumulative link models.

**Table 1.** List of variables included in regression models

Variables	Type	Support
Allocare score	Dependent	Ordinal [1, 2, 3, 4, 5]
Mother religiosity	Predictor	Continuous, scaled
Father religiosity	Predictor	Continuous, scaled
Household proximity	Moderator	Categorical
Allocare category	Moderator (robusticity checks only)	Categorical
Religious group	Moderator (robusticity checks only)	Categorical
Alloparent gender	Moderator (robusticity checks only)	Categorical
Focal child age	Moderator (robusticity checks only)	Categorical
Alloparent category	Moderator (robusticity checks only)	Categorical
Socioeconomic status	Covariate	Continuous, scaled
Mother education	Covariate	Ordinal
Father education	Covariate	Ordinal
Mother age	Covariate	Discrete
Father age	Covariate	Discrete
Allocare task type	Random effect	Categorical
Family ID	Random effect	Categorical
Alloparent ID	Random effect	Categorical
Child ID	Random effect	Categorical

We then fit the ordered logit regressions, regressing allocare frequency on both mother’s and father’s religiosity, and alloparent proximity. Potential confounders identified by the DAG were included as covariates (see above). Spousal pair, focal child, alloparent, and allocare item were included as random effects, in order to account for non-independence between observations within the grouping variables. Separate models were fit for parents’ private religiosity and public religiosity, which tested whether the effects of private or public religiosity on allocare frequency were differentially moderated by the proximity of the alloparent to the parent.

First, we tested for main effects of mother’s and father’s private and public religiosities on allocare. Using likelihood ratio tests, we then tested whether alloparent proximity moderated the effects of mother’s and father’s religiosities on allocare. Finally, we tested interactions between father’s and mother’s religiosity, as well as three-way interactions with alloparent proximity. In subsequent models, we tested the robusticity of the religiosity–allocare relationship by assessing the potentially moderating effects of child age group, parent religion, allocare category, alloparent gender, and alloparent relationship.

### 4.2. Main Findings

***1. Associations between parents’ private religiosity and allocare frequency***

To test for main effects, we regressed the ordinal allocare frequency variable, conditioned on at least some allocare having occurred, on mother’s private religiosity, father’s private religiosity, as well as the prespecified controls and alloparent proximity using a cumulative logit model. There were no significant associations between mother’s (OR = 1.14, *SE* = .09, *p* = .111) or father’s (OR = .89, *SE* = .07, *p* = .171) private religiosity and allocare. See Supplement page S19 for all coefficients from the main effects model.

Next, we tested whether alloparent proximity moderated the effects of mother’s and/or father’s private religiosity on allocare. Three additional regressions were fit, identical to the above, but with one each of the three possible two-way interactions between alloparent proximity, and mother’s and father’s religiosity, respectively. We then compared the interaction models to the baseline model without interactions using likelihood ratio (LR) tests.

**Figure 2.**
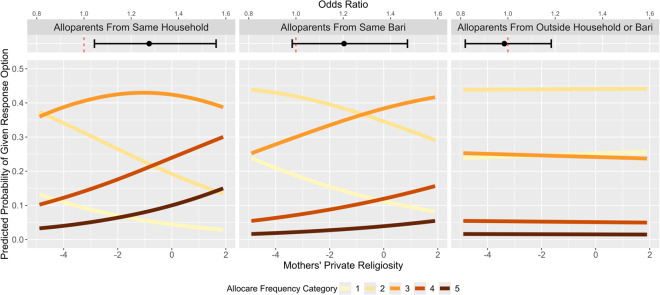
**Associations between private religiosity and allocare.** Plot of simple effects (odds ratios) and predicted probabilities from the ordered logit model in which allocare frequency was regressed on the interaction between alloparent proximity and mother’s private religiosity. Simple effects for mother’s private religiosity at each level of alloparent proximity are plotted at the top of the plot. A predicted probability plot is then plotted below the coefficient for each of the simple effects respectively. Along the *x*-axis of the predicted probability plots is mother’s private religiosity, which is standardized and hence expressed in terms of standard deviations. Note that in the data set, mother’s private religiosity ranged from −4.99 to 1.89. Allocare frequency was measured on a 1–5 ordinal scale, where 1 represents the least frequent amount of allocare and 5 the most frequent. The predicted probabilities of a given allocare frequency response option were then derived from the ordered logit model across the observed range of mother’s private religiosity (using .1 unit increments), and at each level of alloparent proximity. The predicted probability plots thus visualize the predicted response processes underlying the simple effect coefficients, expressed here in odds ratios. For within-household alloparents, where the effect of mother’s private religiosity was significant and positive, that effect was driven by increases in the predicted probability of respondents indicating allocare at frequency categories 4 and 5, and decreases in categories 1 and 2, as mother’s private religiosity increased. In contrast, for outside household alloparents, predicted response options did not change across the range of mother’s private religiosity.

There were no significant interactions between alloparent proximity and father’s private religiosity (LR = .33, df = 2, *p* = .847), and between mother’s and father’s private religiosity (LR = .89, df = 1, *p* = .345). However, there was an interaction between alloparent proximity and mother’s private religiosity (LR = 8.71, df = 2, *p* = .013). We then extracted the associations between mother’s private religiosity and allocare at each level of alloparent proximity. Results indicated that mother’s private religiosity did have a positive association with allocare frequency, but only among alloparents living within the same household as the focal child (see Figure 2). That is, the odds of any given act of allocare being performed more frequently by same-household alloparents were significantly higher among more privately religious mothers. The effect was modest in size (OR = 1.30, 95% CI [1.04, 1.56]), indicating that for every one standard deviation increase in mother’s private religiosity, the odds of receiving allocare more frequently from within-household alloparents increased by 30%.

The effect trended in a similar direction among alloparents from the same *bari*; however, the 95% confidence interval of the odds ratio included one (.98, 1.47). Comparing the simple effects, the difference in estimates between same-household versus outside-bari was statistically significant (OR = 1.30, *SE* = .10, *p* = .023). However, there were no significant differences between same household and same *bari* alloparents (OR = 1.06, *SE* = .11, *p* = .859), or same *bari* and non-same-household/*bari* alloparents (OR = 1.22, *SE* = .09, *p* = .086). Note that *p*-values were adjusted for multiple comparisons using the Tukey method.

Focusing on the effects of mother’s private religiosity on alloparents from the same household, we then probed the robusticity and generalizability of that association across a range of different conditions. To do so, we tested whether the effect of interest was moderated by a series of potential confounds. The positive relationship between allocare frequency and mother’s private religiosity among same-household alloparents was invariant across parent religion (Muslim and Hindu), alloparent gender, type of allocare (indirect and direct), and focal child age group.

**Figure 3.**
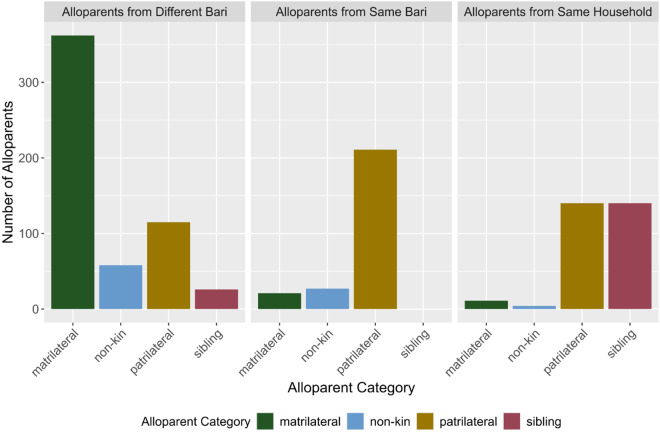
**Frequency of alloparent types across proximity categories.** Plot showing the number of alloparents per relational category across the three proximity levels. Most same-household alloparents were siblings or patrilateral kin of the focal child. Most same-*bari* alloparents were patrilateral kin, with small numbers of matrilateral kin and non-kin. The majority of alloparents from other households or *bari* were matrilateral kin, although the other categories were represented at lower frequencies.

Of particular importance given that the various categories of alloparents (e.g. matrilateral and patrilateral kin) systematically differed across the three proximity categories (see Figure 3), we wanted to rule out the possibility that the effects of proximity were being confounded by the nature of the relationship between the alloparent and focal children. Therefore, we tested whether alloparent relationship moderated the effect of mother’s private religiosity on allocare frequency from same-household alloparents. Across patrilateral kin and siblings – the categories of alloparent commonly found within the household – the effect of mother’s private religiosity on allocare was invariant (see Figure S10 in Supplement). Further, controlling for alloparent relation did not conceptually affect the results (see Supplement page S23). Although this suggests that mother’s private religiosity was similarly affecting patrilateral kin and sibling alloparents within the household – and not patrilateral kin and siblings outside the household – these results do not fully rule out the possibility that the results are confounded by alloparent relationship. For example, matrilateral kin alloparents were very rare within the same household or *bari* (likely owing to patrilocal residence patterns), and as a result, the effects described above could result from systematic differences between matrilateral alloparents on the one hand, and patrilateral/sibling alloparents on the other. The above results were conceptually similar across a variety of other robusticity criteria: when using a less complex model with a simpler random effects structure and without fixed effect controls (Supplement page S23), as well as when using the allocare frequency outcome that included zeroes (Supplement page S23).

We also analysed the effects of private religiosity on the number of alloparents in the focal children’s care networks. As stated in the Methods section, this outcome was treated as exploratory, because the size of an allocare network does not necessarily correspond with the amount of care provided by that network, a metric better captured by the frequency outcome. Nevertheless, network size may be a relevant target for religious signalling, particularly if religiosity has the effect of inducing members of the religious community to provide care. To test this possibility, we modeled a child’s number of alloparents using negative binomial regressions, with the same independent variables and covariates as the models above. Random effects were included for family ID and child ID. In contrast to the positive relationship between mother’s private religiosity and allocare frequency, there was a negative relationship between the number of alloparents and mother’s private religiosity (IRR = .85, *SE* = .03, *p* < .001). This effect was driven by alloparents outside the household (see Supplement page S37). There was no effect of father’s private religiosity on the number of alloparents (see Supplement page S37).

***2. Associations between parents’ public religiosity and allocare frequency***

**Figure 4.**
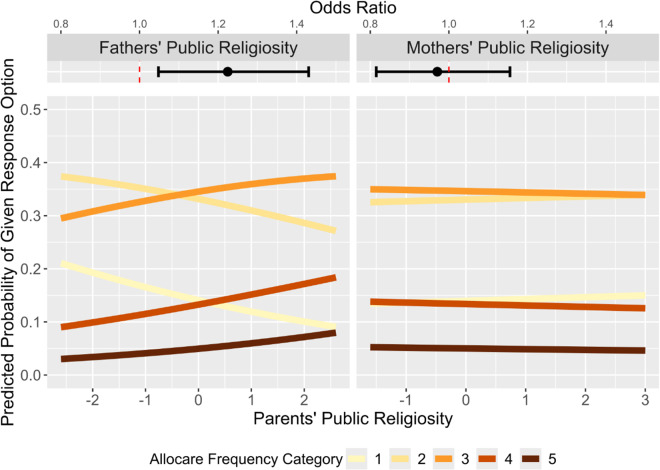
**Effects of parental public religiosity on allocare.** Main effects and predicted probabilities from the ordered logit model in which allocare frequency was regressed on parents’ public religiosity (and additional controls, see Analytical Strategy section). Because there were no significant interactions between public religiosity and alloparent proximity, in contrast to Figure 2, only main effects are plotted. See Figure 2 for a description of the predicted probability plots.

We next tested whether public religiosity would elicit more frequent allocare using the same analytic strategy. However, in comparison to private religiosity, public religious practices are in theory accessible to the majority of co-religionists, whether or not they reside in the same household as the parents. Using the same methodology as above, we first tested the main effects of parental public religiosity on allocare. There was a positive effect of father’s (OR = 1.22, *SE* = .10, *p* = .011), but not mother’s (OR = .97, *SE* = .09, *p* = .737), public religiosity on allocare frequency (see Figure 4). See Supplement page S20 for all coefficients from the main effects model.

We then tested for interactions between parent religiosity and alloparent proximity. Likelihood ratio tests indicated that there were no interactions between alloparent proximity, and either father’s (LR = 10.86, df = 2, *p* = .054) or mother’s (LR = 6.30, df = 2, *p* = .278) public religiosity, respectively. Likewise, there was no significant interaction between spouses’ public religiosity (LR = 7.47, df = 1, *p* = .113). Although the likelihood ratio test indicated that the interaction between father’s public religiosity and alloparent proximity was non-significant, the *p*-value (.054) approached significance. As an exploratory post-hoc analysis, we subsequently assessed the simple effects of father’s public religiosity at each level of alloparent proximity. The effect of father’s public religiosity on alloparents outside the household or *bari* was marginally stronger than the effect on alloparents within the household. See Supplement page S24 for details.

The same generalizability checks as above were then conducted to probe the robusticity of the main effect of father’s public religiosity on alloparenting frequency. There were no moderating effects of age group, religion, alloparent gender, or allocare type on the association between father’s public religiosity and support received from alloparents (see Figure S3). A likelihood ratio test further showed that alloparent relationship did not moderate the effect of father’s public religiosity (LR = 3.24, df = 1, *p* = .072), and controlling for alloparent relationship did not affect the results (see Supplement page S23). For all the above analyses, results were conceptually similar when using a less complex model with a simpler random effects structure and without fixed effect controls (Supplement page S23), as well as when using the allocare frequency outcome that included zeroes (Supplement page S23).

We again analysed the effect of religiosity on the number of alloparents in a child’s care network, using mixed negative binomial regressions. In contrast to the negative relationship between mother’s private religiosity and the number of alloparents, father’s public religiosity positively corresponded with how many people provided their children with care (IRR = 1.11, *SE* = .04, *p* = .006). This was primarily driven by alloparents within the same *bari* as the father (see Supplement page S37). There was no effect of mother’s public religiosity on the number of alloparents (see Supplement page S37).

## 5. Discussion

We found evidence that parents’ religiosity had positive affordances for the allocare received from co-religionists. In particular, when providing allocare, the alloparents of more religious parents tended to provide that care more frequently compared to the alloparents of less religious parents. In contrast to simple dyadic models of cooperation, these results point to the importance of considering complex family units. Further, consistent with the religious allocare hypothesis (Shaver *et al*., 2019), the informational availability of different kinds of religious practices and rituals had moderating effects on the relationship between parent religiosity and alloparental support. Private religious behaviours, such as reciting religious texts within the home, are primarily observable by other members of the household. Alloparents outside the household are less likely to acquire information about the frequency of those private behaviours. Therefore, private religious practices may not carry meaningful signal quality for those alloparents outside the household.

Consistent with this possibility, private religiosity only affected the care provided by alloparents within the household, although this effect only obtained for mothers. In contrast, public religious behaviours could be observed by all alloparents regardless of proximity. Concordantly, public religiosity associated with allocare across household categories. This effect was specific to fathers. Therefore, the effects of observability were moderated by parent gender, such that mother’s and father’s religiosities were delineated in private and public domains, respectively. It is possible that public and private religiosity have different signalling values as a function of proximity. For example, private religious behaviours may primarily be targeted at members of the same household, meaning that prospective within-household alloparents attend more to that information even if they have access to knowledge about parents’ public religious practice as well.

Expanding on the moderating effect of parent gender, mother’s private religiosity influenced same-household alloparents, whereas father’s public religiosity influenced alloparents generally. If some religious rituals hold signal value for putative caregivers, as our results suggest, the observed effects of gender indicate that various social factors may modulate the relevance of different behaviours for those alloparents. Given that in Matlab, men are typically outside the home more often than women, and thus more publicly visible, engagement in public religious ritual may have culturally evolved as a more meaningful signal of religious commitment among men compared to women, which is consistent with prior theory and empirical findings (CaiRangDongZhi, *et al.*, 2023; Sosis & Ruffle, 2004). That is, if women’s public religious behaviour is more circumscribed (as suggested by both the data – see Supplement page S7 – and ethnographic observation), it may not retain as much signal value. Concordantly, private religious ritual may therefore be the primary behavioural output of variation in women’s religious commitment, and thus more important than men’s private religiosity in influencing alloparental care. This is consistent with the fact that Matlab women are more likely to engage in home religious practice (Lynch *et al.*, 2022).

The results are also consistent with the possibility that different kinds of religious rituals have specific signal value for potential caregivers. If the effect of religiosity on alloparental support was merely a by-product of some unaccounted confounding process, then we would not necessarily expect a pattern of results wherein the visibility of the signal moderates its effect. Therefore, the moderating effect of household proximity provides evidence for the signalling mechanism predicted by the religious alloparenting hypothesis. These effects were invariant across several potential moderators, including alloparent gender, allocare types, child age, and religion. Hinduism and Islam differ substantially in their religious beliefs and rituals, yet the above effect obtained among members of both traditions, indicating that these results may generalize across different socioreligious contexts.

Moreover, the composition of alloparents’ relational categories varied systematically across the different proximity categories. We tested whether these differences may be confounding the moderating effect of proximity. Within proximity categories, the results of interest were invariant across the different relational categories of alloparents commonly found in each proximity category, respectively. Further, controlling for alloparent category did not conceptually affect the results. However, we cannot fully rule out a confounding effect of alloparent relation given that some types of alloparents were simply not present in some proximity categories. For example, matrilateral alloparents were extremely common outside the household, and barely present within the household. Differences between outside- and same-household alloparents could be driven by this bias, even if there were no differences between matrilateral and non-matrilateral alloparents within the outside-household category.

However, the positive relationship between religiosity and allocare was not always consistent across different outcomes. Although allocare frequency more closely measures the amount of care received by a child – and hence proxies the benefit obtained by parents for eliciting allocare – religiosity could also act on related outcomes, such as the number of alloparents in a child’s care network. This may be especially true for public religiosity, where larger care networks could be accessed more efficiently via religious signalling. This possibility was borne out by the positive relationship between father’s public religiosity and the number of alloparents providing their children with care. However, results were ultimately mixed, as the children of mothers who engaged in more private practices had fewer alloparents, contrary to predictions. Although frequency may be closer to the proposed mechanism of action linking religiosity with allocare, these results complicate that connection, and suggest that additional research is needed to disambiguate why the relationship would move in opposite directions across these different outcomes.

There are other explanations that could lead to the observed effects of gender. In the above, we posit that there are empirical differences between men’s and women’s engagement in public religious ritual, which potentially influences the signal value of those behaviours for potential alloparents. For example, if women truly engage in fewer public rituals, it may be a weak or noisy signal. However, these results could also be the result of measurement error, for instance if the operationalization of religiosity in our survey fails to capture finer gradations in women’s public practice. Although we attempted to mitigate against this possibility by deriving survey questions from ethnographic focus groups, the possibility remains.

Another possibility that could connect father’s public religiosity with alloparental support broadly is that more observable religious practices may be costlier on average, perhaps due to larger opportunity costs, or the relative benefits of having the costliest practices being witnessed by the largest audience. Likewise, public acts are inherently more likely to be collective, as they typically involve practitioners congregating together, and hence may foster cohesiveness that leads to greater alloparental care. Therefore, these kinds of religious practices may have an outsized effect on receiving aid irrespective of observability per se.

Residence patterns may also contribute to the differential effects of parent gender on received allocare. Specifically, because this society is patrilocal, most alloparents residing within the household are patrilateral relatives of the focal children. Allocare performed by closely related patrilateral relatives may be relatively insensitive to variation in father’s religiosity, if those effects are swamped out by the larger impacts of kin-based cooperation (Crittenden & Marlowe, 2008) and/or non-independence between relatives’ religiosities (Voas & Storm, 2022). In contrast, patrilateral relatives may attend more strongly to their relative’s wife’s religiosity as a signal of either perceived parental quality, and/or quality as a direct cooperator, hence eliciting more alloparental support. Future research ought to tease apart this possibility, and determine whether the effect of gender reverses, by replicating this research in matrilocal societies. Moreover, husbands’ public religiosity did not correlate with their wives’ religiosity, indicating that these were separable signals. If they were isomorphic, we should expect less gender-based variance. Future research should also elucidate whether the moderating effect of gender attenuates in societies where wives’ and husbands’ religious behaviour is more strongly correlated.

Taken in sum, this research is consistent with the religious alloparenting and costly signalling of religion hypotheses (Shaver *et al.*, 2019; Sosis & Alcorta, 2003). Across a religiously diverse community, the care provided by alloparents in Matlab, Bangladesh covaried with the frequency in which the parents benefiting from that care engaged in religious rituals. However, the results also indicate that the religious alloparenting effect is culturally contingent. For example, culturally articulated gender roles and residence patterns appear to have influenced what types of religious rituals were germane to alloparents. Under different societal configurations, the precise effects observed in this study may not replicate. For example, the distinction between private and public religious ritual may vary in importance depending on the size of households, the particular idiosyncratic practices of any given religion, and so on. This indicates the significance of cultural variation interacting with potential adaptations involving allocare and religious signalling.

This research is also limited in important ways. First, the data were cross-sectional in nature, and therefore we cannot conclusively disambiguate the direction of causality. For example, parents who receive more direct allocare may have more free time to engage in non-parenting activities, including religious ritual. However, as noted in the Results section, we did specify a directed acyclic graph in order to partially address potentially biasing paths, including confounders related to wealth, age, and education. More importantly, the moderating effects of household proximity provide evidence for a mechanism connecting religious signalling to care received, which is consistent with the causal direction posited by the religious alloparenting hypothesis.

Second, these results were limited to a single cultural context, albeit one in which people belong to diverse religious traditions. However, given the posited evolved function of religious signalling vis-à-vis alloparental support, it is important to test the cross-cultural consistency and variation in the relationship between religiosity and allocare. On the one hand, we expect that culture will have a substantial effect on structuring the precise nature of gender roles, familial relationships, and ritual categories in shaping when and for whom religiosity elicits allocare. For example, we would not expect the same pattern of results for mother’s versus father’s religiosity to appear universally. Instead, the role of mothers and fathers in eliciting allocare likely varies across cultural contexts. On the other hand, the general principles of the religious allocare hypothesis should obtain across diverse cultural contexts. Thus, while the precise details and configurations may vary, the general tenet that religiosity associates with receiving greater care should be consistent. Further cross-cultural research would help elucidate the accuracy of this hypothesis.

Third, the effect sizes were quite small (Rosenthal, 1996), with the primary effects of interest (expressed in odds ratios) ranging from 1.21 to 1.31. Given the overdetermined nature of both religiosity and cooperative childcare (e.g. Major-Smith *et al.*, 2023; Martin *et al.*, 2020), and the noisiness associated with obtaining estimates of these behaviours in the real world, it is perhaps unsurprising that the effects were not large. However, the small effects indicate that while the religious alloparenting hypothesis may be accounting for some of the variation in alloparental care, many other factors go into determining how much support an alloparent will provide.

Fourth, the survey required that women recollect about the allocare directed at their offspring. Although mother’s assessments of this aid are likely to be fairly accurate – particularly because mothers tend to also stay in the home (Lynch *et al.*, 2022) – there may be various biases that prejudice the accuracy of those assessments. Likewise, there may be a social desirability bias for participants, especially mothers, to claim high religiosity (Presser & Stinson, 1998; Shaver *et al.*, 2021), although we observed relatively wide variation in religiosity (see Figure 1). Direct observation of alloparental care and religious ritual would be a more direct measure of the outcome of interest. Relatedly, in order to facilitate recall for participants, allocare was measured using ordered categories. This made it difficult to systematically sum the total allocare received by focal children.

The study also has several notable strengths. Research on the social effects of religion often treats individuals as isolated units of analysis. However, this potentially minimizes the importance of diffuse familial and/or inclusive benefits in religious networks, as well as the role of culture in shaping familial and gender norms that affect the elicitation and reception of care. Towards that point, in this study, the positive affordances of religiosity for allocare only becomes fully apparent when taking into consideration both spousal partners within families. To our knowledge, this is the first study showing that father’s religiosity positively relates to the amount of allocare received by their children. Whereas prior work has shown such an effect for mothers (Shaver e*t al.*, 2019, 2020, 2024; Spake *et al.*, 2024), these results indicate that the religious alloparenting hypothesis may have culturally contingent, domain-specific effects across gender. That said, there were no meaningful interactions between mother’s and father’s religiosity in eliciting allocare, suggesting that their effects are independent. As suggested in the Introduction, an interdependence between them might indicate that family units are more than the sum of their parts in the context of securing alloparental care; however, we find no evidence of this. In addition, although the data were cross-sectional, the moderating effects of household proximity – in conjunction with variation in the extent to which different religious rituals are publicly performed – provides mechanistic evidence for a causal effect of religiosity on allocare.

Given the universality of religious ritual and alloparenting (Brown, 1991; Sear & Mace, 2008), as well as intense focus on the relationship between religion and fertility (Wormald, 2015), research on the relationship between religiosity and alloparental care is of substantial interest. While the question of whether religion is adaptive remains controversial, this research provides mechanistic evidence from a diverse population supporting the costly signalling theory of religion (Sosis & Alcorta, 2003) generally, and the religious alloparenting hypothesis specifically (Shaver *et al.*, 2019). That religious parents received allocare from their support networks more frequently suggests that religious cooperation may be one avenue for ameliorating the tradeoff between fertility and investment, and hence may in part explain the paradox of religious fertility.

Although allocare is frequently framed as being directed at mothers and their offspring (e.g. Herlosky & Crittenden, 2021), our results indicate that under certain contexts, that care is also being directed at – or at least influenced by – fathers and their inclusive fitness interests. Religious signalling, cooperation, and reciprocity are typically assessed at the individual level – that is, research tests whether more religious individuals accrue more cooperative support. Yet, these effects may be domain specific across societally determined gender roles. Differences in the composition of alloparents across proximity categories suggests that religiosity may affect consanguineal and affinal kin differently, perhaps reflecting divergent fitness interests. These results show that when assessing predictors of alloparental support, both mothers and fathers should be taken into consideration. At a broader level, research on cooperation and religiosity needs to consider complex social units as both signalers of prosocial intent, and receivers of subsequent aid.

## Supporting information

Samore et al. supplementary material Samore et al. supplementary material

## Supplementary material

The supplementary material for this article can be found at https://doi.org/10.1017/ehs.2025.10029.

## Research transparency and reproducibility

The complete questionnaires in English, translations, pre-registrations, and analysis code along with the anonymized data required to replicate the analyses are available at https://osf.io/b865v/.
