# Supplementary material for: Religious parents receive more alloparental aid in rural Bangladesh
Source: Evol Hum Sci. 2025 Dec 12;8:e5. doi: 10.1017/ehs.2025.10029 (PMC12895441; doi:10.1017/ehs.2025.10029)
Supplement: Samore et al. supplementary material [file S2513843X25100297sup001.docx]

**Supplementary Information for**

Religious Parents Receive More Alloparental Aid in Rural Bangladesh

Theodore Samore^*^, Richard Sosis, John Shaver, Radim Chvaja, Matthew Conrad, Anushé Hassan, Robert Lynch, Susan Schaffnit, Rebecca Sear, Laure Spake, Joseph Watts, & Mary Shenk

* Corresponding author: theo.samore@gmail.com

Contents

[1. Differences Between Pre-Registration and Final Manuscript 2](#_Toc199108242)

[2. Scale Construction 3](#_Toc199108243)

[1. Religiosity 3](#_Toc199108244)

[2. SES 13](#_Toc199108245)

[3. Directed Acyclic Graph 15](#_Toc199108247)

[4. Zero-Order Correlations 16](#_Toc199108248)

[5. Regression Equation 18](#_Toc199108249)

[6. Results Supporting Main Text 18](#_Toc199108250)

[1. Full model coefficients 18](#_Toc199108251)

[2. Effects of potential moderators on religiosity-allocare relationship 21](#_Toc199108252)

[3. Controlling for alloparent relationship 23](#_Toc199108253)

[4. Simplified models 23](#_Toc199108254)

[5. Allocare frequency including zeroes 23](#_Toc199108255)

[6. Interaction between fathers’ public religiosity and alloparent proximity 24](#_Toc199108256)

[7. Exploratory Outcomes 34](#_Toc199108257)

[1. Types of care 34](#_Toc199108258)

[2. Number of Alloparents 37](#_Toc199108259)

[8. Packages and Session Info 40](#_Toc199108260)

[9. References 43](#_Toc199108261)

# Differences Between Pre-Registration and Final Manuscript

There were several differences between the pre-registration and final manuscript, as detailed below.

1. In the pre-registration, we planned to assess the relative effects of mothers’ and fathers’ religiosity at the three fieldsites (in Bangaldesh, India, and the Gambia) where data were collected on fathers’ religiosity. However, this plan was changed following publication of the pre-registration. First, we identified a data collection issue in India wherein mothers were only reporting one or two exemplar alloparents, and not their full allocare networks as research assistants had been directed to probe. We realized this made it impossible to assess allocare frequency across a wide range of alloparents. Thus, the Indian data was excluded. We also realized that given the depth of analysis required within each fieldsite, it was unfeasible to include data from both The Gambia and Bangladesh in the same manuscript. Therefore, a decision was made to separate these into two separate manuscripts. Results from The Gambia using these same analyses will be published in a future manuscript.
2. In the pre-registration, we reported that we would used Bayesian mixed models to analyze the data. However, in the final manuscript, we utilized frequentist models instead owing to the computational costs of running ordered logit regressions with multiple random effects across a large number of models.
3. In the pre-registration, we planned to analyze total alloparental care received per child, as well as average allocare received per child per alloparent. However, as explained in the main text, the allocare data were ordinal in nature, and cannot simply be summed or averaged like metric data could be. After publishing the pre-registration, we determined that it would be statistically unadvisable to try and calculate a summed or averaged index of total allocare.
4. In the pre-registration, we planned to analyze the number of types of care provided by each alloparent as an outcome. However, upon further reflection, it was not clear whether providing more types of care constitutes greater helpfulness in the absence of frequency data. We therefore treat this outcome as exploratory. Likewise, the number of alloparents in a child’s network may or may not reflect help received in the absence of information about frequency, and was also treated as exploratory. See Exploratory Outcomes section of Supplement for results with these outcomes. There was an additional outcome reported in the pre-registration based on the probability of receiving allocare from different types of alloparents (e.g., maternal grandparents, siblings, etc.). Because this particular manuscript was not focused on these fine-grained categories of alloparents, we reserve this outcome for future research.

# Scale Construction

## Religiosity

Principal component analyses were conducted to create public and private religiosity scores for mothers and fathers. Because religiosity differs across both gender roles and religion, principal component analyses were conducted separately for men and women, and Hindus and Muslims respectively. Therefore, religiosity scores were relative to other people of the same gender and religion.

For each principal component analysis, we first separated the religiosity items into public religious behaviors (behaviors that occur outside the household, e.g. attending worship services), private religious behaviors (behaviors that occur inside the household, e.g. reciting religious texts), and subjective reports of religiosity (e.g. self-identification of religiosity relative to other community members). No further analysis was conducted on the subjective religiosity items since this study was concerned with the effects of religious behavior on eliciting alloparental help.

As alluded to above, the specific items varied across religion and gender (although some items were held in common). For example, Hindus were asked Hindu-specific questions such as whether they participate in Ekadosi fasting, while Muslims were asked whether they fast during Ramadan. Further, some items were ordinal in nature (e.g., “do you recite rudraksh mantara”, on a three-point ordinal scale from “never” to “always”), while others were counts of activities (e.g., “what activities do you perform at home: reading/reciting religious text, praying…”) or frequencies (e.g., how many times in the past 4 weeks did you attend temple/mosque for prayer or to listen to religious discussion, etc.?”).

Note that some items were excluded if they were at ceiling or floor (for example, nearly all Muslim fathers in the sample reported praying five times a day). Count variables were log-transformed. The items that were included in the principal component analyses for each combination of public and private religiosity, gender, and religion are listed below. Note that these items were translated into Bengali for participants, see https://osf.io/b865v/ for full surveys with translations.

1. Private religiosity – Muslim Women:
   1. How many obligatory prayers do you perform daily? (ordinal):
      1. none
      2. Sometimes
      3. once per day
      4. twice per day
      5. three times per day
      6. four times per day
      7. five times per day
   2. Do you perform the obligatory fasting during the month of Ramadan? (ordinal)
      1. Never
      2. Sometimes
      3. Always
   3. Outside of Salat, how frequently do you recite from the Holy Quran? (ordinal)
      1. Never
      2. Sometimes
      3. Always
   4. What [private religious activities] do you perform at home? (count)
      1. Reading/reciting religious text
      2. Praying
      3. Visiting religious websites/youtube
      4. Reading books about religion
      5. Watching religious programs on TV/listening to religious programs on the radio
      6. other
   5. How frequently do you involve yourself in [the religious activities performed at home]? (ordinal)
      1. Daily
      2. Weekly
      3. Monthly
      4. 3-4 times a year
      5. Yearly
2. Public religiosity – Muslim Women:
   1. Do you visit distant places for religious discussions? (ordinal)
      1. Never
      2. Sometimes
      3. Always
   2. How many times in the past 4 weeks did you attend temple/mosque for prayer or to listen to religious discussion, etc.? (count)
   3. How many times in the past 4 weeks did you attend any religious events, discussion, or other gathering somewhere in your bari or a relative/neighbor’s bari? (frequency)
   4. What kinds of activities did you participate in/attend in the past 4 weeks? (count)
      1. None
      2. Faith-based charity/volunteering
      3. Reading groups
      4. Prayer groups
      5. Women’s group
3. Private religiosity – Hindu Women:
   1. What [private religious activities] do you perform at home?
   2. How frequently do you involved yourself in [the religious activities performed at home]?
   3. How many times do you recite Geeta at your house? (ordinal)
      1. One time per day
      2. At least one time per week
      3. At least one time per month
      4. Rarely
   4. Which of the holy books do you ever recite? (count)
      1. Vogobot
      2. Geeta
      3. Bed
      4. Uponishod
   5. Do you follow the below mentioned rules as a Hindu / [which of the following do you do]? (count)
      1. Water a basil plant in the house
      2. Ekadosi fasting
      3. Perform batshorik pooja and saptahik pooja
      4. Not eat beef
      5. Maintain the quality of being holy
4. Public religiosity – Hindu Women:
   1. How many times in the past 4 weeks did you attend temple/mosque for prayer or to listen to religious discussion, etc.?
   2. How many times in the past 4 weeks did you attend any religious events, discussion, or other gathering somewhere in your bari or a relative/neighbor’s bari?
   3. What kinds of activities did you participate in/attend in the past 4 weeks? (count)
   4. Do you visit distant places for religious discussions?
   5. How many times do you go to Mandir to pray? (ordinal)
      1. One time per day
      2. At least one time per week
      3. At least one time per month
      4. Rarely
      5. Never
5. Private religiosity – Muslim Men:
   1. Do you perform the obligatory fasting during the month of Ramadan?
   2. Outside of Salat, how frequently do you recite from the Holy Quran?
   3. What [private religious activities] do you perform at home?
   4. How frequently do you involve yourself in [the religious activities performed at home]?
6. Public religiosity – Muslim Men:
   1. How many times in the past 4 weeks did you attend temple/mosque for prayer or to listen to religious discussion, etc.?
   2. How many times in the past 4 weeks did you attend any religious events, discussion, or other gathering somewhere in your bari or a relative/neighbor’s bari?
   3. How many obligatory prayers do you perform daily
7. Private religiosity – Hindu Men:
   1. What [private religious activities] do you perform at home?
   2. How frequently do you involve yourself in [the religious activities performed at home]?
   3. Which of the holy books do you ever recite?
   4. How many times do you recite Geeta at your house?
   5. Do you follow the below mentioned rules as a Hindu / [which of the following do you do]? (count)
      1. Water a basil plant in the house
      2. Ekadosi fasting
      3. Perform batshorik pooja and saptahik pooja
      4. Do sondhya bati/sajher bati in the evening at your house
      5. Not eat beef
8. Public religiosity – Hindu Men:
   1. Do you visit distant places for religious discussions?
   2. How many times do you go to Mandir to pray?
   3. How many times in the past 4 weeks did you attend temple/mosque for prayer or to listen to religious discussion, etc.?
   4. Do you follow the below mentioned rules as a Hindu? (count)
      1. Marking of sandal paste on forehead and neck
      2. Basil garland on neck
      3. Wearing shakha
      4. Putting vermilion on forehead
      5. Red threat in hand

Before conducting the principal component analyses, we plotted the descriptive statistics for each of the constituent variables (separated by gender and religion), in order to visualize the actual variation in religious practices across individuals, genders, and religions. Likert plots were used for ordinal items, and bar charts were used for count variables. See Figures S1-S4.

**
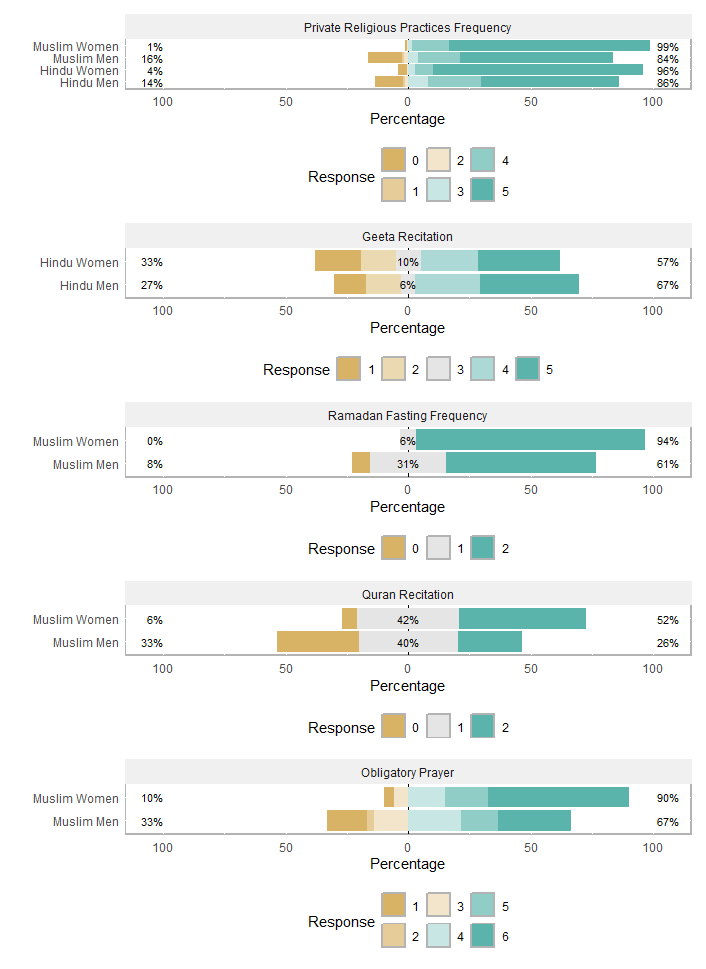
**

**Figure S1 Ordinal Private Religious Practices.** Likert plot showing responses to the individual private religiosity items. Note that some items, such as Quran recitation, were religion specific. Further note that, based on ethnographic observation, obligatory prayer was counted as public religiosity among men, but as private religiosity among women, since men tend to pray in public, and women tend to pray in the home.

**Figure S2 Ordinal Public Religious Practices.** Likert plot showing responses to the individual public religiosity items. Note that some items were religion and/or gender specific. Further note that, based on ethnographic observation, obligatory prayer was counted as public religiosity among men, but as private religiosity among women, since men tend to pray in public, and women tend to pray in the home.


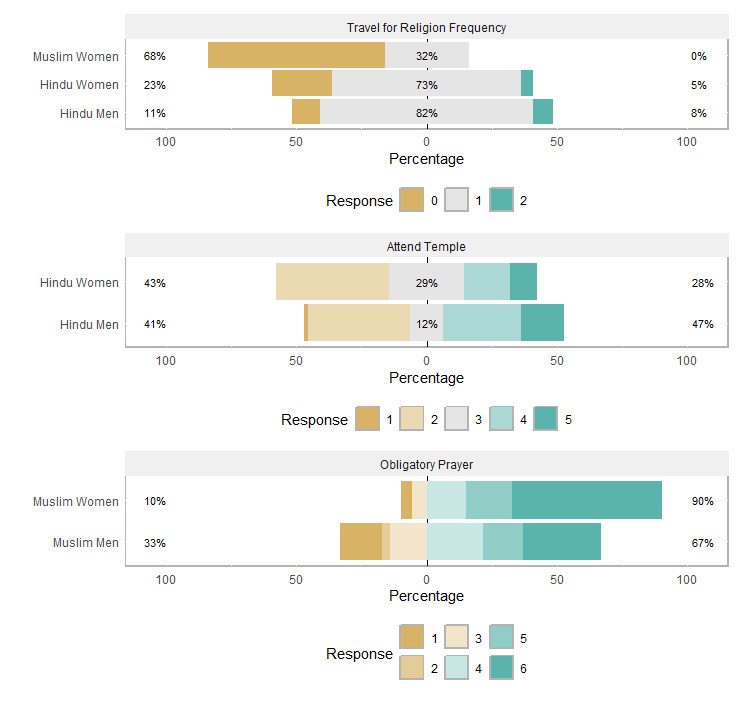


**
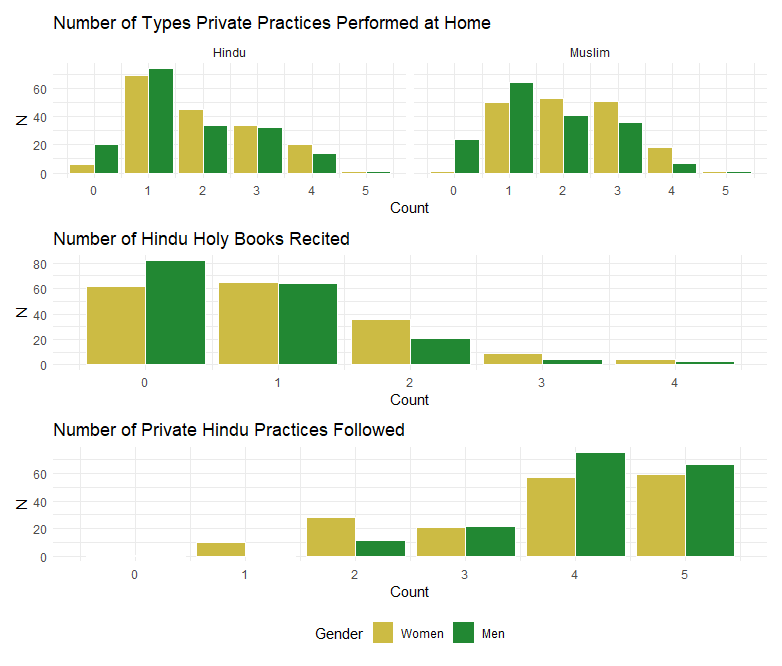
Figure S3 Frequency Private Practices.** Chart showing responses to the private religious practices measured on a frequency scale. Refer to items above for specific practices that were counted (e.g. Ekadosi fasting).

**
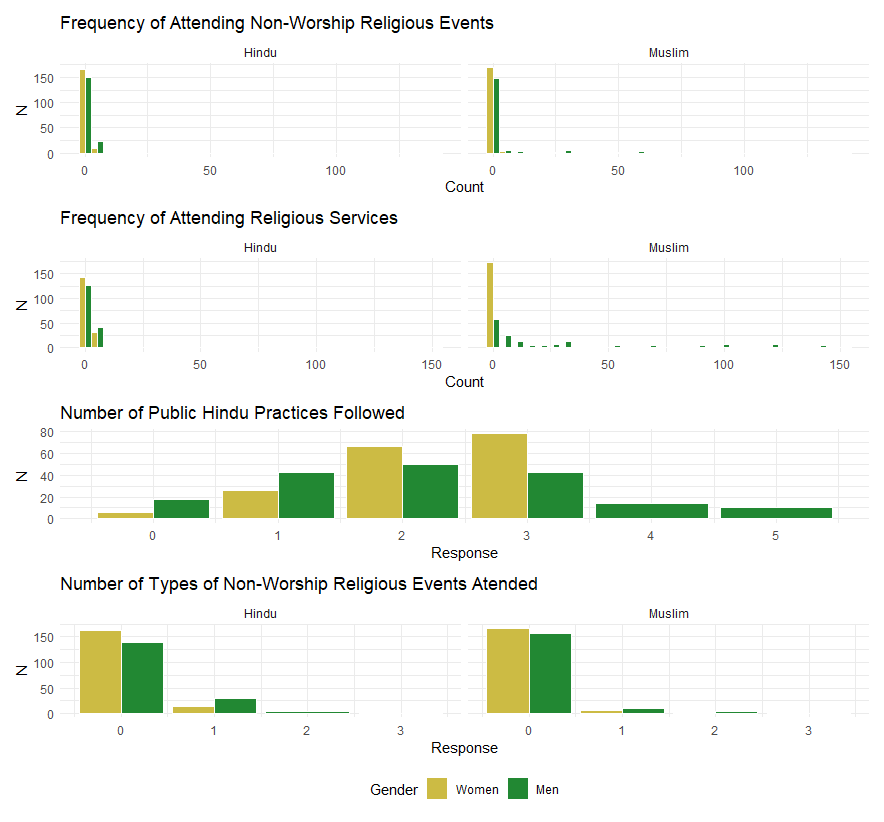
**

**Figure S5 Frequency Public Practices.** Chart showing responses to the public religious practices measured on a frequency scale. Refer to items above for specific practices that were counted.


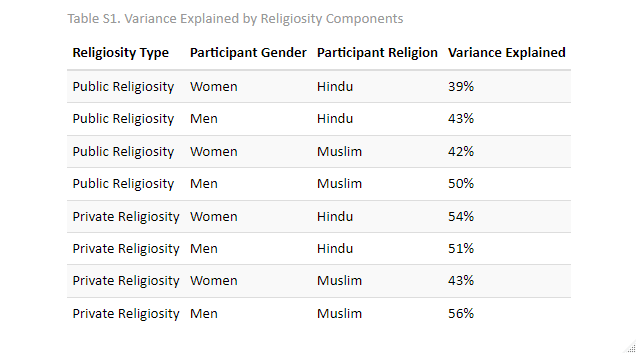
Proceeding with the PCA, within each of these eight groupings, we then calculated correlation matrices. Polychoric correlations were used to calculate the association between two ordered factors, Pearson correlations were used to calculate the association between two log-transformed count variables, and polyserial correlations were used to calculate the association when the variable types differed. A principal components analysis with a single component was then performed on each of the correlation matrices for private and public religiosity, men and women, and Muslims and Hindus respectively. Table S1 shows the variance explained by the public and private religiosity components for the above combinations. Table S2 shows component loadings for each variable.

**Table S2.** Component loadings for each religiosity PCA

| Variable | Muslim Women - Private | Muslim Women - Public | Muslim Men - Private | Muslim Men - Public | Hindu Women - Private | Hindu Women - Public | Hindu Men - Private | Hindu Men - Public |
| --- | --- | --- | --- | --- | --- | --- | --- | --- |
| Private Religious Practices Frequency | 0.55 | - | 0.91 | - | 0.72 | - | 0.72 | - |
| Geeta Recitation | - | - | - | - | 0.78 | - | 0.71 | - |
| Ramadan Fasting Frequency | 0.66 | - | 0.29 | - | - | - | - | - |
| Quran Recitation | 0.75 | - | 0.66 | - | - | - | - | - |
| Obligatory Prayer | 0.70 | - | - | 0.67 | - | - | - | - |
| Travel for Religion Frequency | - | 0.74 | - | - | - | 0.55 | - | 0.58 |
| Attend Temple | - | - | - | - | - | 0.70 | - | 0.79 |
| Number of Private Practices | 0.60 | - | 0.94 | - | 0.89 | - | 0.87 | - |
| Number of Holy Books Recited | - | - | - | - | 0.71 | - | 0.66 | - |
| Number of Private Hindu Practices | - | - | - | - | 0.55 | - | 0.56 | - |
| Frequency of Attending Misc. Events | - | 0.64 | - | 0.69 | - | 0.58 | - | - |
| Frequency of Attending Services | - | 0.32 | - | 0.77 | - | 0.79 | - | 0.77 |
| Number of Public Hindu Practices | - | - | - | - | - | - | - | 0.41 |
| Types of Religious Activities | - | 0.78 | - | - | - | 0.43 | - | - |

## SES

SES was measured as a composite based on a principal component analysis. The following seven variables were included in the analysis: mothers’ ratings of how wealthy and educated their families were relative to their neighbors during childhood (ordinal scale from less wealth/educated to more wealth/educated); mothers’ ratings of how their families’ statuses compared to others in their community during childhood (ladder rating from 1 to 10); ratings of mothers’ families’ wealth and status currently (using the same scales as above); social status of husbands’ jobs (measured on a three-point ordinal scale from least to most status); and a log-transformed measure of household’s objective current wealth.

First, we descriptively plotted each of the seven variables (see figures S6 and S7). Ordinal variables were rated on three-point scales from 2 (more educated/wealthy/etc.) to 0 (less educated/wealthy/etc.). Overall relative status was measured on a continuous scale from 1 to 10, using a ladder metaphor, and total wealth was calculated as the logged sum of all household assets (rescaled to 1 to 10 to match the relative status items).


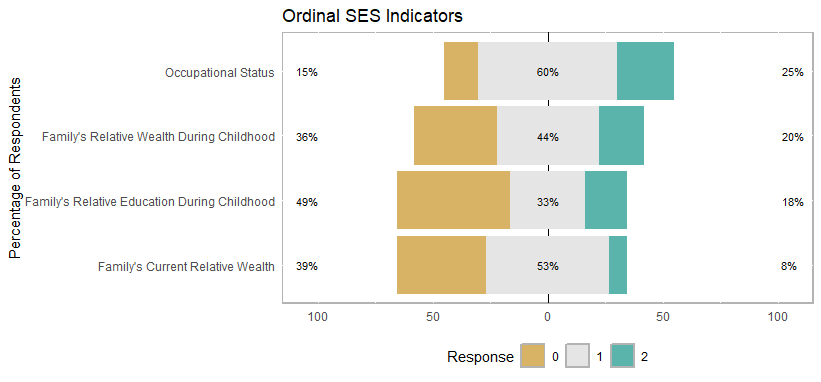
**Figure S6 Ordinal SES Items.** Descriptive plot of the ordinal items used in the SES PCA.


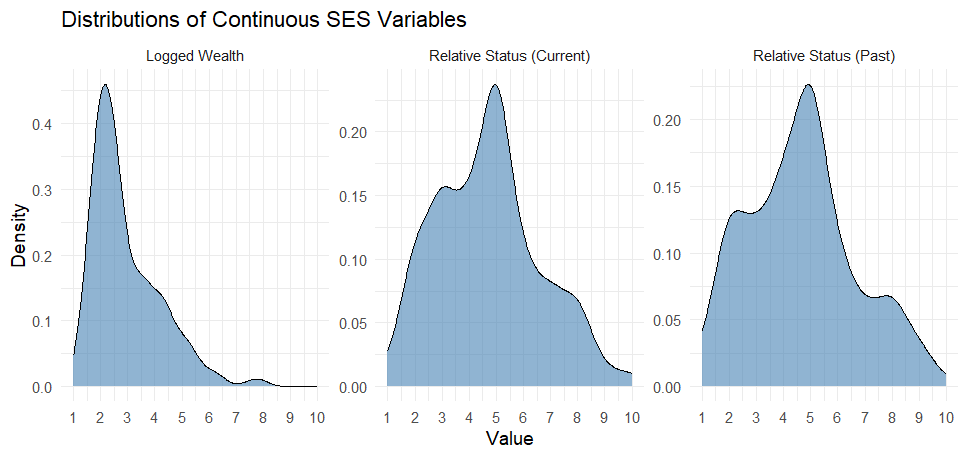


**Figure S7 Continuous SES Items.** Descriptive plot of the ordinal items used in the SES PCA.

We then calculated a correlation matrix for these variables. Polychoric correlations were used to calculate the association between two ordered factors, Pearson correlations were used to calculate the association between two log-transformed count variables, and polyserial correlations were used to calculate the association when the variable types differed. A principal components analysis with a single component was then performed. The resultant SES component explained 46% of the total variance (see Table S3 for item loadings). The standardized component scores were then extracted.

# **Table S3.** Component Loadings for SES PCA

| **Item** | **Factor Loading** |
| --- | --- |
| Occupational Status | 0.42 |
| Family's Relative Education During Childhood | 0.74 |
| Family's Relative Wealth During Childhood | 0.72 |
| Family's Current Relative Wealth | 0.64 |
| Relative Status (Past) | 0.85 |
| Logged Wealth | 0.52 |
| Relative Status (Current) | 0.78 |

# Directed Acyclic Graph

In order to identify variables that may have biased the hypothesized relationship between religiosity and allocare, we pre-registered a directed acyclic graph (see Figure S1). Assuming the causal structure diagrammed in Figure S1, in order to unbiasedly assess the direct relationship between religiosity and allocare, we controlled for parent age, parent education, and household SES.
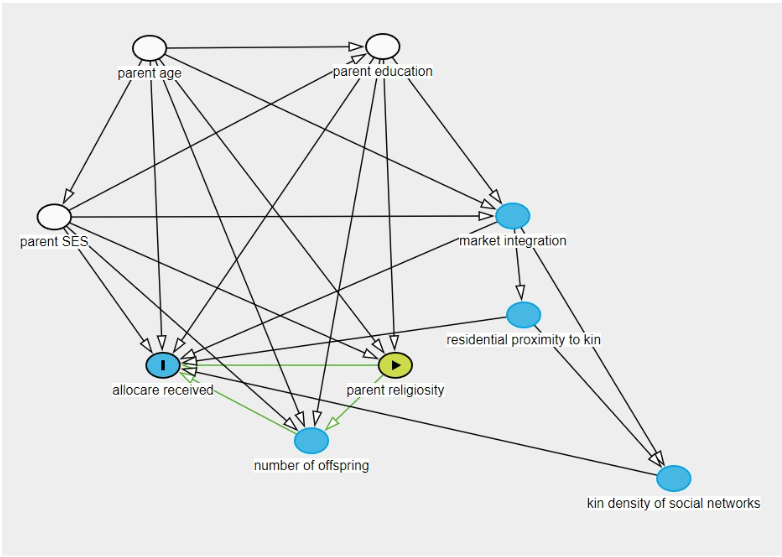


**Figure S8.** Directed Acyclic Graph for relationship between religiosity and allocare.

# Zero-Order Correlations

In the Main Text, we refer to a table with zero-order correlations between the variables reported in the Main Text. Here, we show the zero-order correlations between mothers’ and fathers’ private and public religiosities, and the five covariates (Figure S2). Polychoric correlations were used to calculate the association between ordinal variables (allocare and mothers’ and fathers’ education), Pearson correlations were used to calculate the association between discrete variables (religiosity composites, SES, and mothers’ and fathers’ ages), and polyserial correlations were used to calculate the association when the variable types differed.


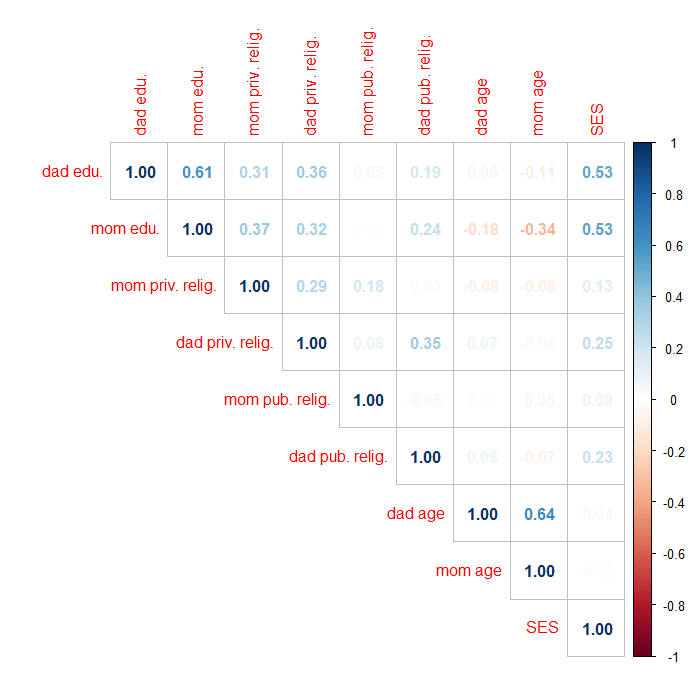


**Figure S9.** Zero-order correlations between independent variables and covariates.

# Regression Equation

In the Main Text, we report providing a general equation for the cumulative link mixed models used in the study. In a cumulative link mixed model, we model the probability of the observed frequency of an allocare act *(Y)* being less than or equal to [1,2,3,4,5] (*k)*. This probability is calculated for each individual allocare event that was observed (*i)*, for each child in our dataset *(j)*. The probability is determined by a linear combination of both fixed and random effects transformed by the inverse logit function. Formally:

$$P\left( Y_{ij}\leq k \right)=\frac{1}{1+\exp\left( -\eta_{ij} \right)}$$

Where $\eta ij$:

$$-\eta ij=\theta k-Xij\beta+uj$$

Where $\theta k$​ are the intercepts for each possible frequency category (except 5), $\beta$ is a vector of coefficients for fixed effects, such as parent religiosity and covariates, and $\mu j$ is the random effects. Interpreting the model coefficients is a matter of taking the logit of $P\left( Yij\leq k \right)$.

# Results Supporting Main Text

## Full model coefficients

In the Main Text, we claimed that full model results were provided for the main effect models for private and public religiosity respectively. Here, we provide those results (Tables S2-S5).


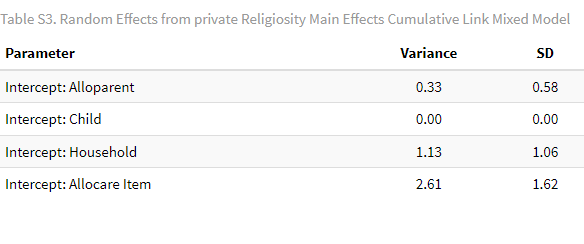

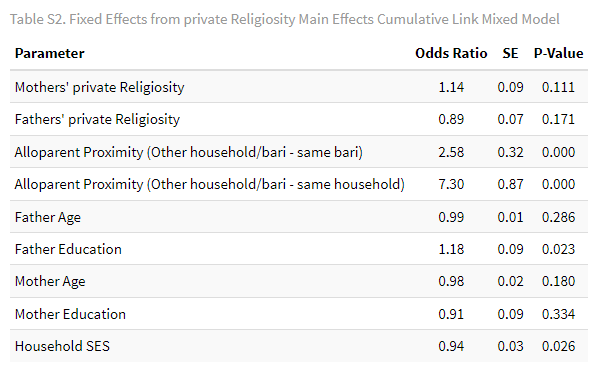


**Table S5.** Random effects from private religiosity main effects model

**Table S4.** Fixed effects from private religiosity main effects model


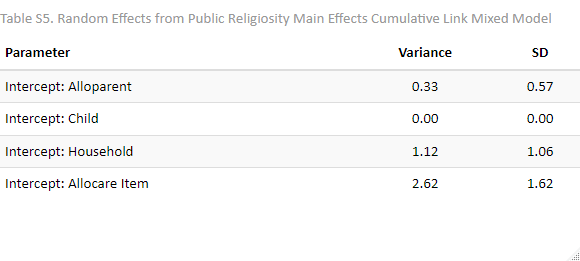

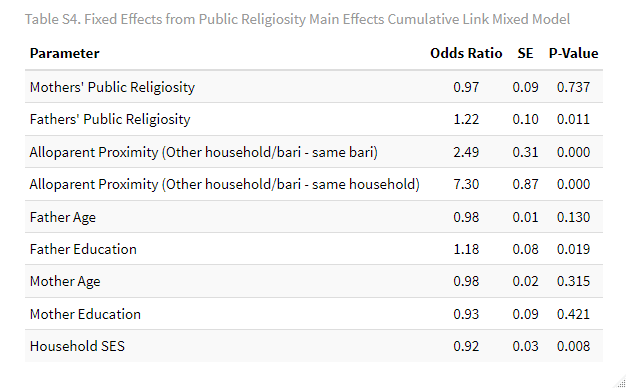


**Table S7.** Random effects from public religiosity main effects model

**Table S6.** Fixed effects from public religiosity main effects model

## Effects of potential moderators on religiosity-allocare relationship

In the Main Text, we report that the positive relationships between allocare frequency and mothers’ private religiosity among same-household alloparents, as well as the relationship between allocare and fathers’ public religiosity, were invariant across parent religion, alloparent gender, type of allocare, focal child age group, and alloparent relationship. To test for invariance, we used the following procedure. For mothers’ private religiosity, we tested the three-way interactions between mothers’ private religiosity, alloparent proximity, and parent religion, alloparent gender, type of allocare, and age group respectively. We additionally tested the two-way interaction between mothers’ private religiosity and matrilateral versus patrilateral same-household alloparents. For fathers’ public religiosity, we tested the two-way interactions with alloparent proximity, parent religion, alloparent gender, type of allocare, age group, and alloparent relationship respectively. Across all models, we used the same allocare frequency outcome, random effects, and cumulative link models used in the primary Main Text analyses. See Figure S3 for results.

**
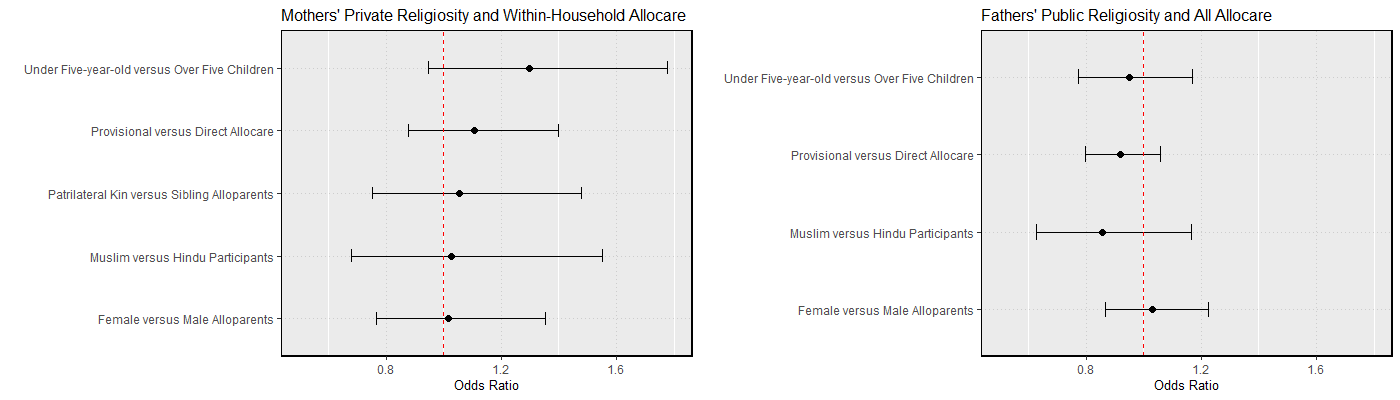
**

**Figure S10. Generalizability of religiosity-allocare relationship.** Plot depicting pairwise comparisons between simple effects when allocare is regressed on the interactions between parental religiosity and the following variables respectively: focal child age group; allocare type; alloparent relationship; participant religion; and alloparent gender. These variables were used to test the robusticity of the observed effects across a range of different conditions. The plot aggregates pairwise comparisons from a series of models used to test each potential moderator individually. Here, we focus on the main findings of interest: the effect of mothers’ private religiosity on within-household allocare (left panel), and the effect of fathers’ public religiosity on allocare broadly (right panel). Note that for the left panel, we only tested the pairwise comparison between the religiosity effects for patrilateral alloparents versus sibling alloparents because these were the only categories that were commonly found within the household (see Figure 3 in Main Text). For the right panel, because all four categories of alloparent were observed at appreciable frequencies among alloparents outside the household/*bari*, in lieu of pairwise comparisons, a likelihood ratio test was used to assess the interaction (see Results section in Main Text). Because of the large number of permutations between alloparent proximity, fathers’ and mothers’ private and public religiosities, and these various moderator variables, we only tested these moderators on the primary effects of interest identified in the Main Text.

## Controlling for alloparent relationship

In the Main Text, we report that controlling for alloparent relationship did not conceptually affect the main results. To test this, we replicated the primary Main Text models, adding a parameter for alloparent relationship.

First, there was no main effect of fathers’ private religiosity on allocare (see Table S8 for model summary). However, there was an interaction between mothers’ private religiosity and alloparent proximity (LR = 13.20, df = 2, *p* = .001). Examining the model with the interaction (see Table S9 for simple effects), there was a positive relationship between mothers’ private religiosity and allocare frequency among same-household alloparents, but not alloparents in the same *bari* or outside the household/*bari*.

Likewise, there was a main effect of fathers’ public religiosity on allocare frequency (see Table S10 for model summary), but not of mothers. There was no interaction between fathers’ public religiosity and alloparent proximity (LR = 1.59, df = 2, *p* = .452).

## Simplified models

In the Main Text, we report that using models with a simpler random effects structure (random intercepts for household ID and allocare item only) and without controls did not conceptually affect the main results. We conducted these analyses to assess whether results were sensitive to the inclusion of covariates and/or the complex random effects structure. There was no main effect of fathers’ private religiosity on allocare (see Table S11 for model summary). However, there was an interaction between mothers’ private religiosity and alloparent proximity (LR = 9.87, df = 2, *p* = .007), such that there was a positive relationship between mothers’ private religiosity and allocare frequency among same-household alloparents, but not alloparents in the same *bari* or outside the household/*bari* (see Table S12 for simple effects).

Likewise, there was a main effect of fathers’ public religiosity on allocare frequency, but not of mothers (see Table S13 for model summary). There was no interaction between fathers’ public religiosity and alloparent proximity (LR = 5.60, df = 2, *p* = .061).

## Allocare frequency including zeroes

As explained in the Main Text, the data on allocare frequency was zero-inflated. That is, most alloparents did not perform any acts of allocare for most of the allocare categories across the specified timeframe. Further, the processes that generated zeroes are plausibly distinct from the processes that contributed to performing certain types of allocare more frequently. For example, aspects of the alloparent including gender or age may contribute to the types of care they provide, but not the frequency at which they perform types of care that are part of their social portfolio. However, on an exploratory basis, we tested whether including zeroes would conceptually affect the main results reported in the Main Text. Results were as follows.

The effect of parents’ private religiosity on allocare frequency was conceptually unaffected compared to results reported in the Main Text, although some effect sizes were smaller. There was no main effect of fathers’ private religiosity on allocare (see Table S14 for model summary). However, there was an interaction between mothers’ private religiosity and alloparent proximity (LR = 13.20, df = 2, *p* = .001), such that there was a positive relationship between mothers’ private religiosity and allocare frequency among same-household alloparents, but not alloparents in the same *bari* or outside the household/*bari* (see Table S15 for simple effects).

Likewise, there was a main effect of fathers’ public religiosity on allocare frequency, but not of mothers (see Table S16 for model summary). There was no interaction between fathers’ public religiosity and alloparent proximity (LR = 1.59, df = 2, *p* = .452).

## Interaction between fathers’ public religiosity and alloparent proximity

In the Main Text, we reported that there was a non-significant trend in the interaction between fathers’ public religiosity and alloparent proximity, and that we assessed this interaction on a post-hoc exploratory basis. Here, we present those results. The effect of fathers’ public religiosity on alloparents outside the household or *bari* was marginally stronger than the effect on alloparents within the household. The effects on same *bari* alloparents did not significant differ from the effects on outside household/*bari* or within household alloparents. See Table S17 for simple effects and pairwise comparisons.

**Table S8.** Summary of fixed and random effects when regressing allocare on private religiosity, controlling for alloparent relationship.


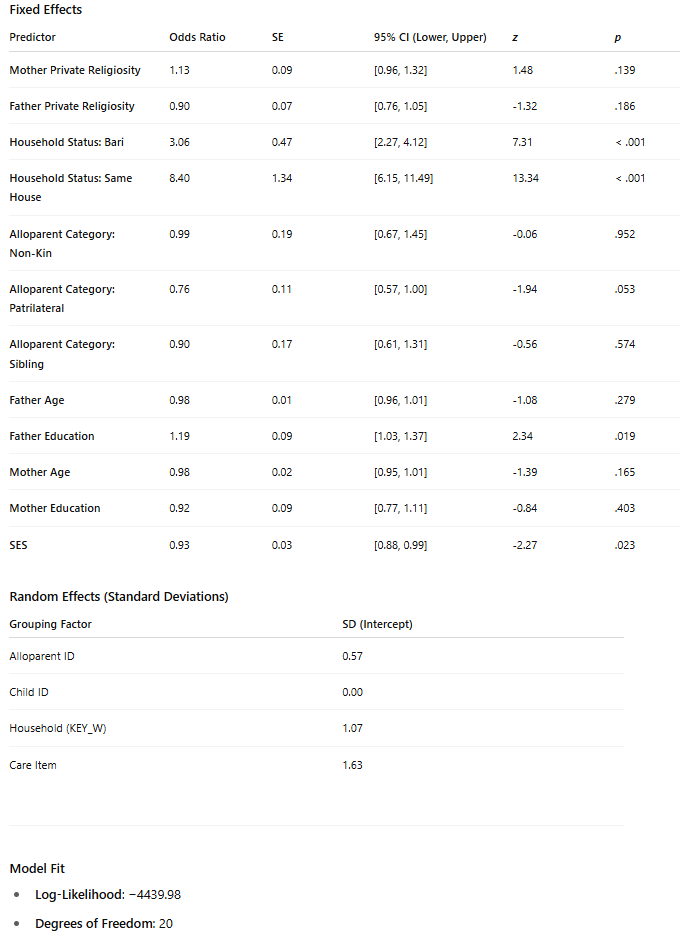


**Table S9.** Simple effects of mothers’ private religiosity on allocare by household proximity, controlling for alloparent relationship


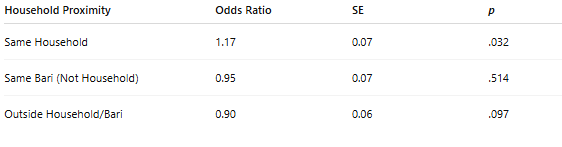


**Table S10.** Summary of fixed and random effects when regressing allocare on public religiosity, controlling for alloparent relationship.


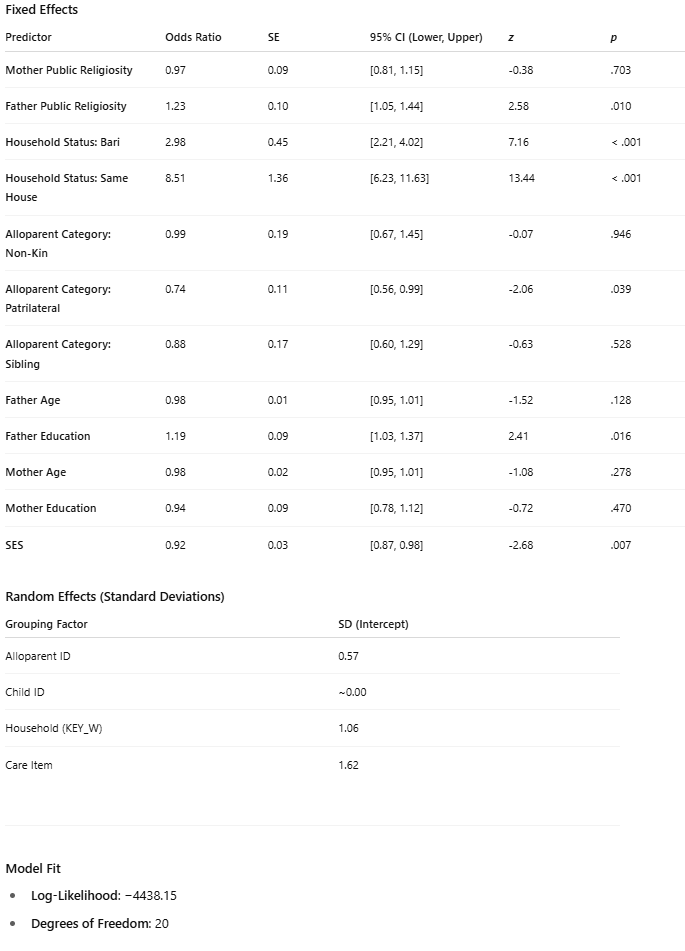


**Table S11.** Summary of fixed and random effects when regressing allocare on private religiosity in a simplified model without covariates.


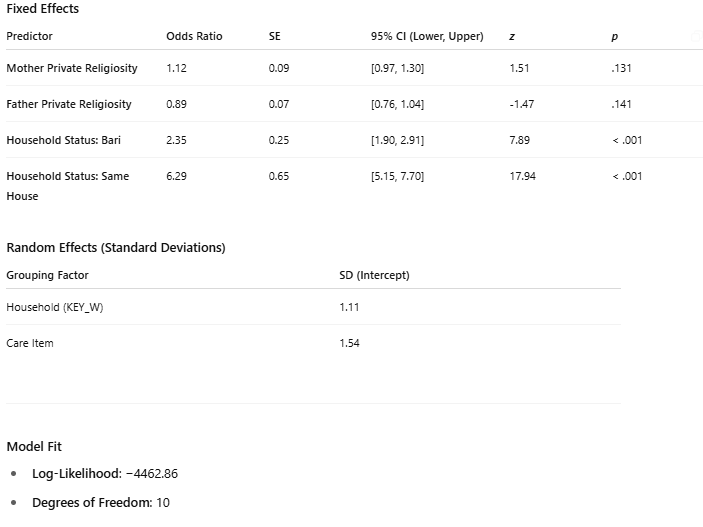


**Table S12.** Simple effects of mothers’ private religiosity on allocare by household proximity in a simplified model without covariates


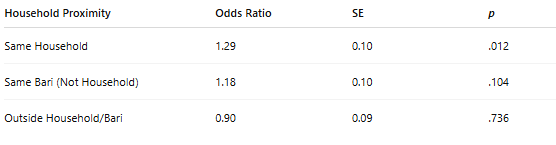


**Table S13.** Summary of fixed and random effects when regressing allocare on public religiosity in a simplified model without covariates.


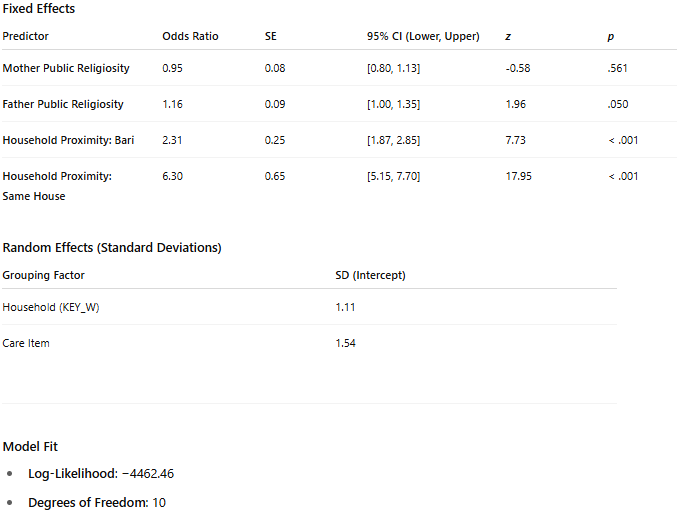


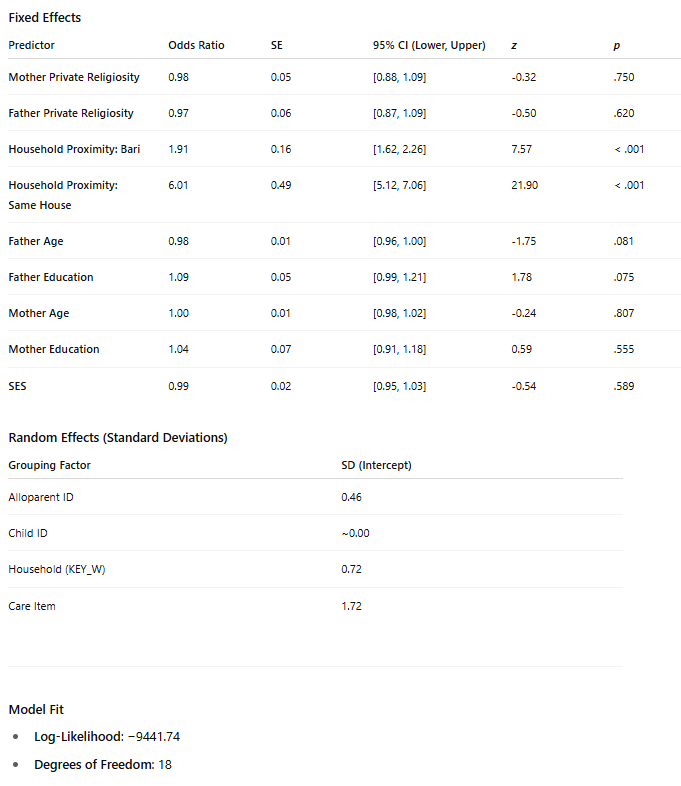
**Table S14.** Summary of fixed and random effects when regressing allocare on private religiosity, including observations of no allocare.

**Table S15.** Simple effects of mothers’ private religiosity on allocare by household proximity, including observations of no allocare


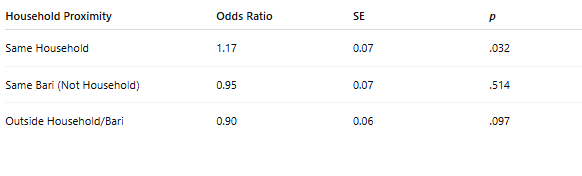


**Table S16.** Summary of fixed and random effects when regressing allocare on public religiosity,


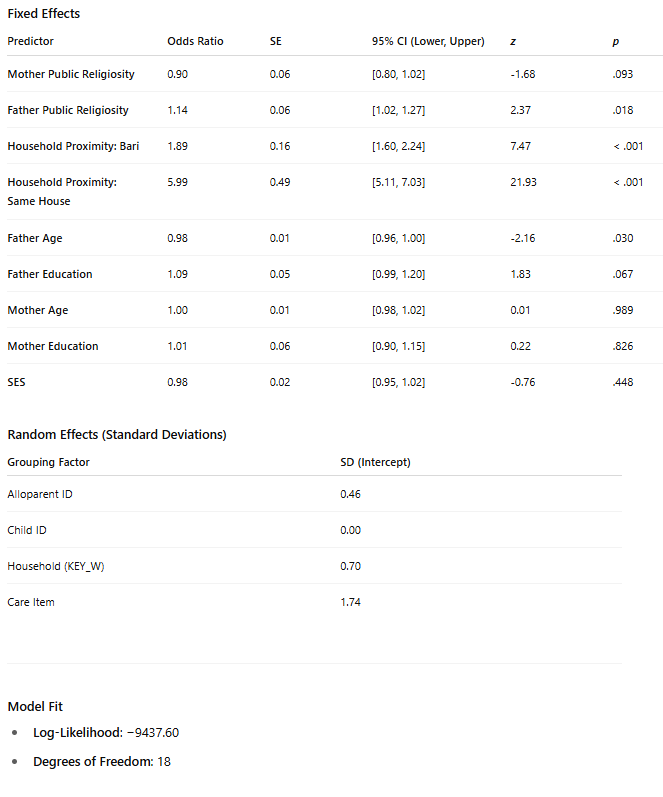
including observations of no allocare.

**Table S17.** Simple effects and pairwise comparisons of fathers’ public religiosity on allocare frequency as a function of household proximity.


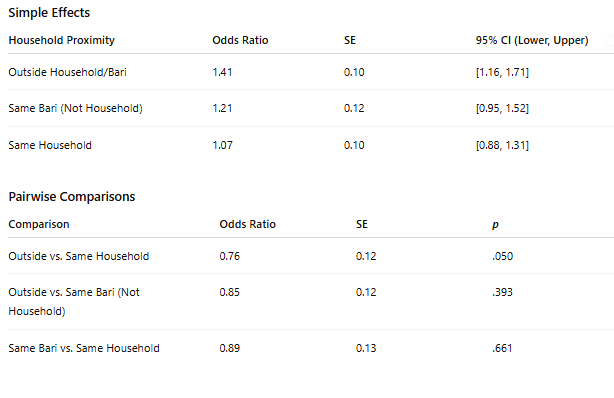


# Exploratory Outcomes

In the Main Text, we report that several alternative outcomes to allocare frequency were considered on an exploratory basis. Those results are presented here.

## Types of care

Another way to handle the zero-inflated data is to run essentially a hurdle model, where we initially model the odds of allocare being provided at least once in the specified time period across each type of allocare across each alloparent. This also corresponds to the “probability of child receiving care for each allocare item” outcome reported in the pre-registration. However, as explained above, we considered this outcome exploratory given that helping on a greater number of different types of allocare may not correspond with alloparent helpfulness. Here, we report the exploratory results.

In order to test this outcome, for each possible type of allocare for each alloparent, we created a binary variable corresponding to whether at least some care was provided or not. We then modeled this outcome with binomial logistic regressions using the same set of independent variables, covariates, and random effects as in the ordinal models.

There were no main effects of fathers’ or mothers’ private religiosity on the likelihood of a particular allocare act being performed at least once (see Table S18 for model summary). However, there was an interaction between mothers’ private religiosity and alloparent proximity (χ2 = 8.57, df = 2, *p* = .014).

However, the simple effects revealed a different relationship between allocare odds and mothers’ private religiosity relative to allocare frequency. Specifically, there was a negative relationship between mothers’ private religiosity and allocare odds among same *bari* (OR = .85, *SE* = .08, *p* = .039) and different household/*bari* (OR = .86, *SE* = .07, *p* = .025) alloparents, but no relationship among same household alloparents (OR = 1.08, *SE* = .08, *p* = .344). That is, the alloparents of more privately religious mothers outside the household provided fewer types of care (independent of the frequency at which those acts were performed).

For public religiosity, there was a positive main effect of fathers’ public religiosity on allocare odds, but a negative main effect of mothers’ public religiosity (see Table S19 for a model summary). There were no significant interactions between alloparent proximity and either fathers’ (χ2 = 5.42, df = 2, *p* = .067) or mothers’ (χ2 = 3.44, df = 2, *p* = .179) public religiosity.

In sum, it appears that more religious mothers have alloparents who perform fewer types of allocare, although the allocare they do provide is performed more often. In contrast, the alloparents of more publicly religious fathers both perform a greater array of care types, and provide those actions more often.

**Table S18.** Summary of fixed and random effects from a binomial logistic regression in which the number of types of care was regressed on private religiosity, and additional covariates.


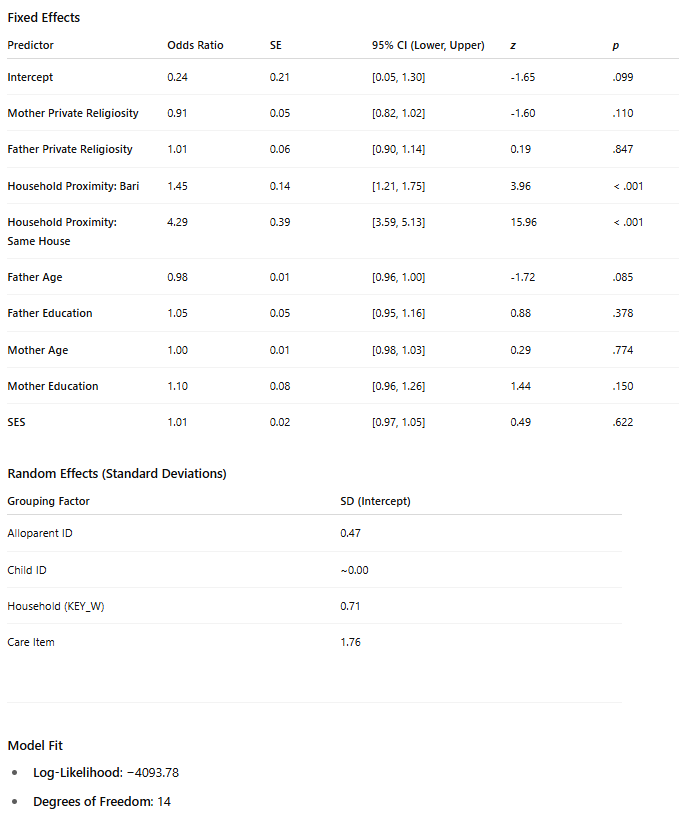


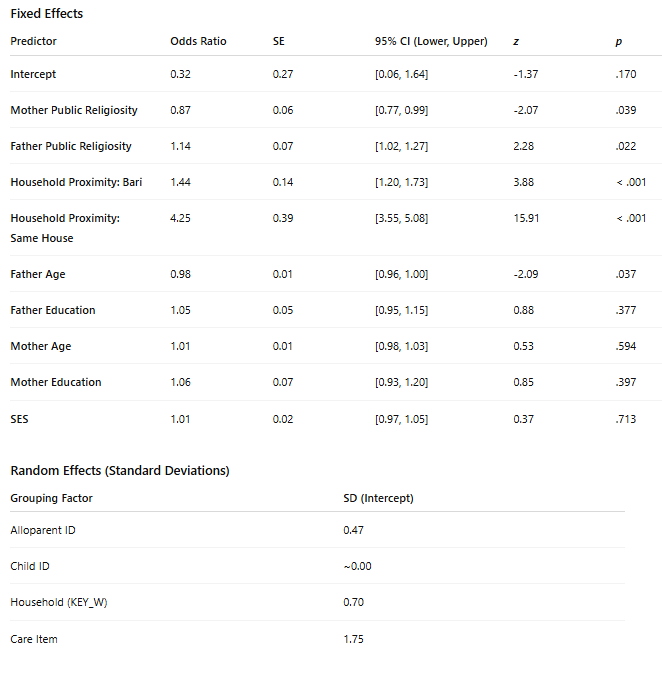
**Table S19.** Summary of fixed and random effects from a binomial logistic regression in which the number of types of care was regressed on public religiosity, and additional covariates.

## Number of Alloparents

As discussed in the Main Text, we also analyzed the number of alloparents in a child’s care network as an exploratory outcome. Below, we provide more details on the models used and their results.

In order to test the alloparent count outcome, we modeled a child’s number of alloparents using negative binomial regressions, with the same independent variables and covariates as the allocare frequency models. Because alloparent count is at the focal child level, and not at the level of individual allocare acts, we included random effects for family ID and child ID. In order to test for the potentially moderating effect of alloparent proximity, we lengthened the data such that there were three observations per child, corresponding to the number of alloparents inside the household, inside the *bari*, and outside the household/*bari*.

There was a negative main effect of mothers’ private religiosity on the number of alloparents in their children’s networks, and no effect of fathers’ private religiosity (see Table 20 for model summary).

There was an interaction between mothers’ private religiosity and alloparent proximity (χ2 = 16.93, df = 2, *p* = .0002), such that the negative relationship between religiosity and alloparent count obtained for same *bari* (IRR = .74, *SE* = .06, *p* < .000) and outside household/*bari* (IRR = .83, *SE* = .04, *p* < .000) alloparent counts, but not the number of alloparents within the household (IRR = .94, *SE* = .05, *p* = .186).

In contrast, there was a positive effect of fathers’ public religiosity on the number of alloparents providing care for his focal children, but no effect of mothers’ public religiosity (see Table 21 for model summary).

There was an interaction with alloparent proximity (χ2 = 12.65, df = 2, *p* = .002), such that more publicly religious fathers had more alloparents within the same *bari* (IRR = 1.34, *SE* = .07, *p* < .000)*,* but no difference in the number of alloparents within the household (IRR = 1.07, *SE* = .05, *p* = .150) or outside the household/*bari* (IRR = 1.07, *SE* = .05, *p* = .132).

**Table S20.** Summary of fixed and random effects from a negative binomial regression in which the number of alloparents in a child’s care network was regressed on private religiosity, and additional covariates.


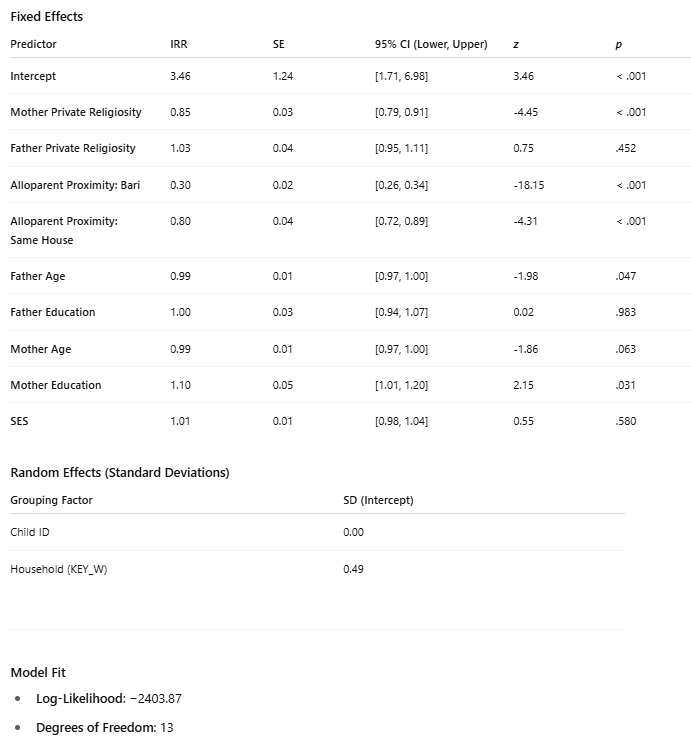


**Table S20.** Summary of fixed and random effects from a negative binomial regression in which the number of alloparents in a child’s care network was regressed on private religiosity, and additional covariates.


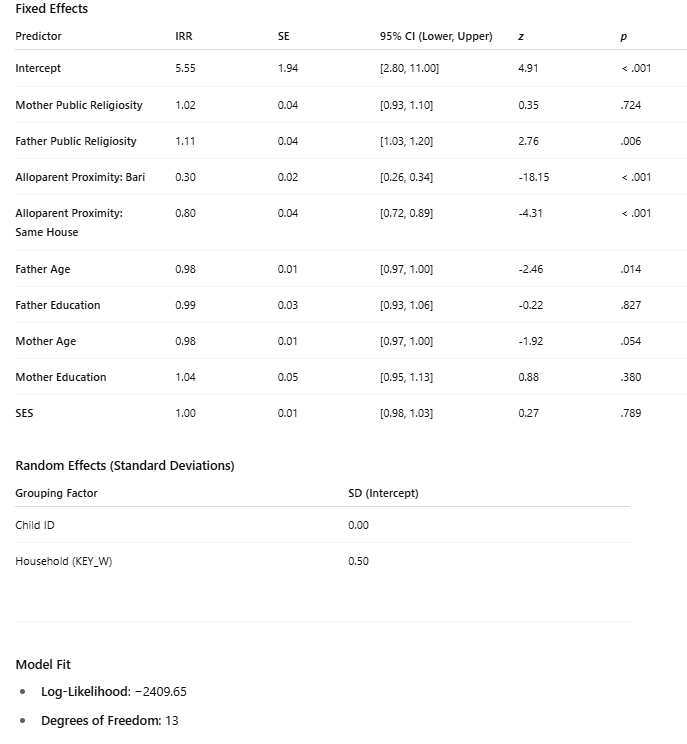


# Packages and Session Info

We used the following software and packages to analyze the data: naniar (Tierney et al., 2024), datawizard (Patil et al., 2022), correlation (Makowski et al., 2023), corrplot (Wei & Simko, 2021), sjPlot (Lüdecke, 2024), effectsize (Ben-Shachar et al., 2020), forcats (Wickham, 2023a), interactions (Long, 2021), emmeans (Lenth, 2024), lubridate (Spinu et al., 2023), Matrix (Bates et al., 2024), patchwork (Pedersen, 2024), psych (Revelle, 2024), performance (Lüdecke et al., 2021), FactoMineR (Lê et al., 2008), see (Lüdecke et al., 2021), tidyr (Wickham et al., 2024), ggdist (Kay, 2024), stringr (Wickham, 2023b), ggExtra (Attali & Baker, 2023), dplyr (Wickham et al., 2023), modelbased (Makowski et al., 2024), knitr (Xie, 2014, 2015), gridExtra (Auguie, 2017), report (Makowski et al., 2023), ordinal (Christensen, 2023), factoextra (Kassambara & Mundt, 2020), kableExtra (Zhu, 2024), tibble (Müller & Wickham, 2023), easystats (Lüdecke et al., 2022), parameters (Lüdecke et al., 2024), ggeffects (Lüdecke, 2018), polycor (Fox, 2022), tidyverse (Wickham, 2023c), ggplot2 (Wickham et al., 2024), readr (Wickham et al., 2024), bayestestR (Makowski et al., 2024), lme4 (Bates et al., 2024), ggridges (Wilke, 2024), insight (Lüdecke et al., 2019), purrr (Wickham & Henry, 2023), mice (Buuren & Groothuis-Oudshoorn, 2011), modelsummary (Arel-Bundock, 2022).

The software and package versions and source information can be found below:


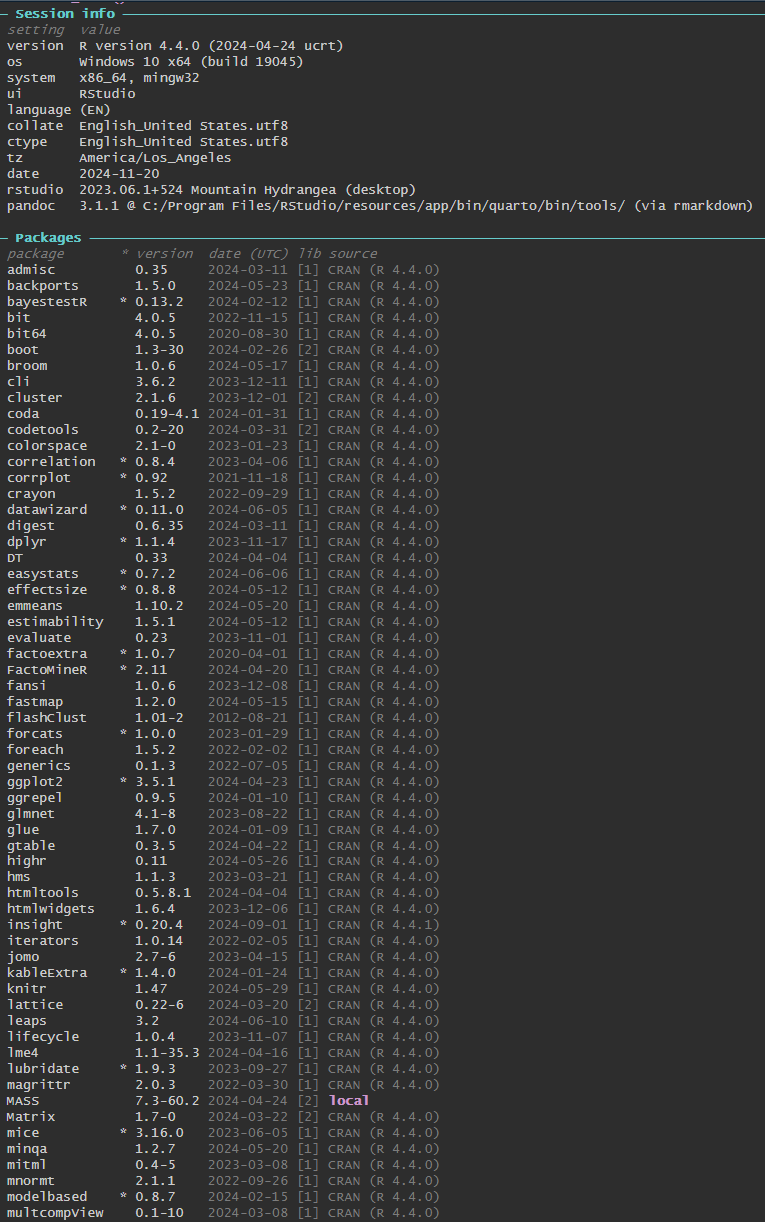


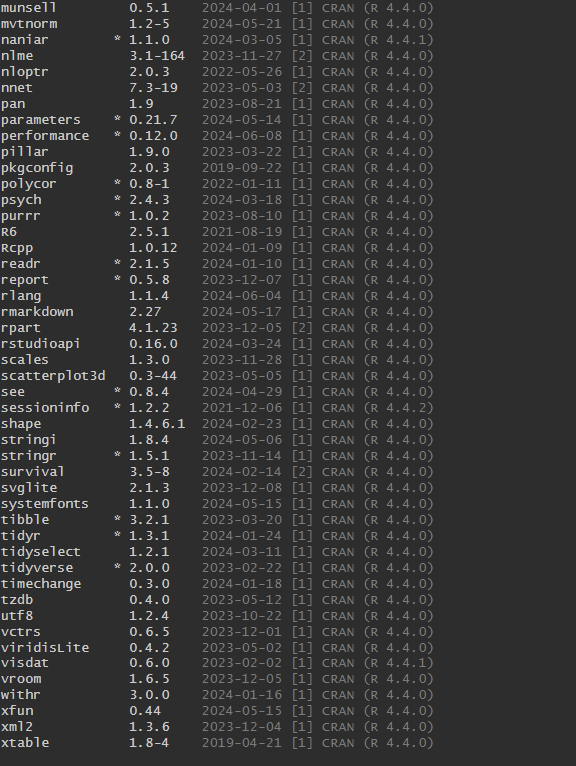


# References

Arel-Bundock, V. (2022). modelsummary: Data and Model Summaries in R. *Journal of Statistical Software*, *103*(1), 1–23. <https://doi.org/10.18637/jss.v103.i01>

Attali, D., & Baker, C. (2023). *ggExtra: Add Marginal Histograms to ggplot2, and More ggplot2 Enhancements*. <https://github.com/daattali/ggExtra>

Auguie, B. (2017). *gridExtra: Miscellaneous Functions for “Grid” Graphics*. <https://CRAN.R-project.org/package=gridExtra>

Bates, D., Maechler, M., Bolker, B., & Walker, S. (2024). *lme4: Linear Mixed-Effects Models using Eigen and S4*. <https://github.com/lme4/lme4/>

Bates, D., Maechler, M., & Jagan, M. (2024). *Matrix: Sparse and Dense Matrix Classes and Methods*. <https://Matrix.R-forge.R-project.org>

Ben-Shachar, M. S., Lüdecke, D., & Makowski, D. (2020). effectsize: Estimation of Effect Size Indices and Standardized Parameters. *Journal of Open Source Software*, *5*(56), 2815. <https://doi.org/10.21105/joss.02815>

Buuren, S. van, & Groothuis-Oudshoorn, K. (2011). mice: Multivariate Imputation by Chained Equations in R. *Journal of Statistical Software*, *45*(3), 1–67. <https://doi.org/10.18637/jss.v045.i03>

Christensen, R. H. B. (2023). *ordinal: Regression Models for Ordinal Data*. <https://github.com/runehaubo/ordinal>

Fox, J. (2022). *polycor: Polychoric and Polyserial Correlations*. <https://r-forge.r-project.org/projects/polycor/>

Grolemund, G., & Wickham, H. (2011). Dates and Times Made Easy with lubridate. *Journal of Statistical Software*, *40*(3), 1–25.

Kassambara, A., & Mundt, F. (2020). *factoextra: Extract and Visualize the Results of Multivariate Data Analyses*. <http://www.sthda.com/english/rpkgs/factoextra>

Kay, M. (2024). ggdist: Visualizations of Distributions and Uncertainty in the Grammar of Graphics. *IEEE Transactions on Visualization and Computer Graphics*, *30*(1), 414–424. <https://doi.org/10.1109/TVCG.2023.3327195>

Lê, S., Josse, J., & Husson, F. (2008). FactoMineR: A Package for Multivariate Analysis. *Journal of Statistical Software*, *25*(1), 1–18. <https://doi.org/10.18637/jss.v025.i01>

Lenth, R. V. (2024). *emmeans: Estimated Marginal Means, aka Least-Squares Means*. <https://rvlenth.github.io/emmeans/>

Long, J. A. (2021). *interactions: Comprehensive, User-Friendly Toolkit for Probing Interactions*. <https://interactions.jacob-long.com>

Lüdecke, D. (2018). ggeffects: Tidy Data Frames of Marginal Effects from Regression Models. *Journal of Open Source Software*, *3*(26), 772. <https://doi.org/10.21105/joss.00772>

Lüdecke, D. (2024). *sjPlot: Data Visualization for Statistics in Social Science*. <https://strengejacke.github.io/sjPlot/>

Lüdecke, D., Ben-Shachar, M. S., Patil, I., & Makowski, D. (2020). Extracting, Computing and Exploring the Parameters of Statistical Models using R. *Journal of Open Source Software*, *5*(53), 2445. <https://doi.org/10.21105/joss.02445>

Lüdecke, D., Ben-Shachar, M. S., Patil, I., Waggoner, P., & Makowski, D. (2021). performance: An R Package for Assessment, Comparison and Testing of Statistical Models. *Journal of Open Source Software*, *6*(60), 3139. <https://doi.org/10.21105/joss.03139>

Lüdecke, D., Ben-Shachar, M. S., Patil, I., Wiernik, B. M., Bacher, E., Thériault, R., & Makowski, D. (2022). easystats: Framework for Easy Statistical Modeling, Visualization, and Reporting. *CRAN*. <https://easystats.github.io/easystats/>

Lüdecke, D., Makowski, D., Ben-Shachar, M. S., Patil, I., Højsgaard, S., & Wiernik, B. M. (2024). *parameters: Processing of Model Parameters*. <https://easystats.github.io/parameters/>

Lüdecke, D., Patil, I., Ben-Shachar, M. S., Wiernik, B. M., Waggoner, P., & Makowski, D. (2021). see: An R Package for Visualizing Statistical Models. *Journal of Open Source Software*, *6*(64), 3393. <https://doi.org/10.21105/joss.03393>

Lüdecke, D., Waggoner, P., & Makowski, D. (2019). insight: A Unified Interface to Access Information from Model Objects in R. *Journal of Open Source Software*, *4*(38), 1412. <https://doi.org/10.21105/joss.01412>

Makowski, D., Ben-Shachar, M. S., Patil, I., & Lüdecke, D. (2020a). Estimation of Model-Based Predictions, Contrasts and Means. *CRAN*. <https://github.com/easystats/modelbased>

Makowski, D., Ben-Shachar, M. S., Patil, I., & Lüdecke, D. (2020b). Methods and Algorithms for Correlation Analysis in R. *Journal of Open Source Software*, *5*(51), 2306. <https://doi.org/10.21105/joss.02306>

Makowski, D., Lüdecke, D., Ben-Shachar, M. S., & Patil, I. (2024). *modelbased: Estimation of Model-Based Predictions, Contrasts and Means*. <https://easystats.github.io/modelbased/>

Makowski, D., Lüdecke, D., Ben-Shachar, M. S., Patil, I., Wilson, M. D., & Wiernik, B. M. (2024). *bayestestR: Understand and Describe Bayesian Models and Posterior Distributions*. <https://easystats.github.io/bayestestR/>

Makowski, D., Lüdecke, D., Patil, I., Thériault, R., Ben-Shachar, M. S., & Wiernik, B. M. (2023). Automated Results Reporting as a Practical Tool to Improve Reproducibility and Methodological Best Practices Adoption. *CRAN*. <https://easystats.github.io/report/>

Makowski, D., Wiernik, B. M., Patil, I., Lüdecke, D., & Ben-Shachar, M. S. (2023). *correlation: Methods for Correlation Analysis*. <https://easystats.github.io/correlation/>

Müller, K., & Wickham, H. (2023). *tibble: Simple Data Frames*. <https://tibble.tidyverse.org/>

Patil, I., Makowski, D., Ben-Shachar, M. S., Wiernik, B. M., Bacher, E., & Lüdecke, D. (2022). datawizard: An R Package for Easy Data Preparation and Statistical Transformations. *Journal of Open Source Software*, *7*(78), 4684. <https://doi.org/10.21105/joss.04684>

Pedersen, T. L. (2024). *patchwork: The Composer of Plots*. <https://patchwork.data-imaginist.com>

R Core Team. (2024). *R: A Language and Environment for Statistical Computing*. R Foundation for Statistical Computing. <https://www.R-project.org/>

Revelle, W. (2024). *psych: Procedures for Psychological, Psychometric, and Personality Research*. <https://personality-project.org/r/psych/>

RStudio Team. (2019). *RStudio: Integrated Development Environment for R*. RStudio, Inc. <http://www.rstudio.com/>

Spinu, V., Grolemund, G., & Wickham, H. (2023). *lubridate: Make Dealing with Dates a Little Easier*. <https://lubridate.tidyverse.org>

Tierney, N., & Cook, D. (2023). Expanding Tidy Data Principles to Facilitate Missing Data Exploration, Visualization and Assessment of Imputations. *Journal of Statistical Software*, *105*(7), 1–31. <https://doi.org/10.18637/jss.v105.i07>

Tierney, N., Cook, D., McBain, M., & Fay, C. (2024). *naniar: Data Structures, Summaries, and Visualisations for Missing Data*. <https://github.com/njtierney/naniar>

Wei, T., & Simko, V. (2021). *R package “corrplot”: Visualization of a Correlation Matrix*. <https://github.com/taiyun/corrplot>

Wickham, H. (2023a). *forcats: Tools for Working with Categorical Variables (Factors)*. <https://forcats.tidyverse.org/>

Wickham, H. (2023b). *stringr: Simple, Consistent Wrappers for Common String Operations*. <https://stringr.tidyverse.org>

Wickham, H. (2023c). *tidyverse: Easily Install and Load the Tidyverse*. <https://tidyverse.tidyverse.org>

Wickham, H., Averick, M., Bryan, J., Chang, W., McGowan, L. D., François, R., Grolemund, G., Hayes, A., Henry, L., Hester, J., Kuhn, M., Pedersen, T. L., Miller, E., Bache, S. M., Müller, K., Ooms, J., Robinson, D., Seidel, D. P., Spinu, V., … Yutani, H. (2019). Welcome to the tidyverse. *Journal of Open Source Software*, *4*(43), 1686. <https://doi.org/10.21105/joss.01686>

Wickham, H., Chang, W., Henry, L., Pedersen, T. L., Takahashi, K., Wilke, C., Woo, K., Yutani, H., Dunnington, D., & Brand, T. van den. (2024). *ggplot2: Create Elegant Data Visualisations Using the Grammar of Graphics*. <https://ggplot2.tidyverse.org>

Wickham, H., François, R., Henry, L., Müller, K., & Vaughan, D. (2023). *dplyr: A Grammar of Data Manipulation*. <https://dplyr.tidyverse.org>

Wickham, H., & Henry, L. (2023). *purrr: Functional Programming Tools*. <https://purrr.tidyverse.org/>

Wickham, H., Hester, J., & Bryan, J. (2024). *readr: Read Rectangular Text Data*. <https://readr.tidyverse.org>

Wickham, H., Vaughan, D., & Girlich, M. (2024). *tidyr: Tidy Messy Data*. <https://tidyr.tidyverse.org>

Wilke, C. O. (2024). *ggridges: Ridgeline Plots in ggplot2*. <https://wilkelab.org/ggridges/>

Xie, Y. (2014). knitr: A Comprehensive Tool for Reproducible Research in R. In V. Stodden, F. Leisch, & R. D. Peng (Eds.), *Implementing Reproducible Computational Research*. Chapman and Hall/CRC.

Xie, Y. (2015). *Dynamic Documents with R and knitr* (2nd ed.). Chapman and Hall/CRC. <https://yihui.org/knitr/>

Zhu, H. (2024). *kableExtra: Construct Complex Table with kable and Pipe Syntax*. <http://haozhu233.github.io/kableExtra/>
